# Uncertainty Quantification Reveals the Importance of Data Variability and Experimental Design Considerations for *in Silico* Proarrhythmia Risk Assessment

**DOI:** 10.3389/fphys.2017.00917

**Published:** 2017-11-21

**Authors:** Kelly C. Chang, Sara Dutta, Gary R. Mirams, Kylie A. Beattie, Jiansong Sheng, Phu N. Tran, Min Wu, Wendy W. Wu, Thomas Colatsky, David G. Strauss, Zhihua Li

**Affiliations:** ^1^Division of Applied Regulatory Science, Center for Drug Evaluation and Research, Office of Translational Sciences, Office of Clinical Pharmacology, Food and Drug Administration, Silver Spring, MD, United States; ^2^Centre for Mathematical Medicine and Biology, School of Mathematical Sciences, University of Nottingham, Nottingham, United Kingdom; ^3^Marshview Life Science Advisors, Seabrook Island, SC, United States

**Keywords:** uncertainty quantification, experimental variability, cardiac electrophysiology, action potential, Torsade de Pointes, ion channel, pharmacology, computational modeling

## Abstract

The Comprehensive *in vitro* Proarrhythmia Assay (CiPA) is a global initiative intended to improve drug proarrhythmia risk assessment using a new paradigm of mechanistic assays. Under the CiPA paradigm, the relative risk of drug-induced Torsade de Pointes (TdP) is assessed using an *in silico* model of the human ventricular action potential (AP) that integrates *in vitro* pharmacology data from multiple ion channels. Thus, modeling predictions of cardiac risk liability will depend critically on the variability in pharmacology data, and uncertainty quantification (UQ) must comprise an essential component of the *in silico* assay. This study explores UQ methods that may be incorporated into the CiPA framework. Recently, we proposed a promising *in silico* TdP risk metric (qNet), which is derived from AP simulations and allows separation of a set of CiPA training compounds into Low, Intermediate, and High TdP risk categories. The purpose of this study was to use UQ to evaluate the robustness of TdP risk separation by qNet. Uncertainty in the model parameters used to describe drug binding and ionic current block was estimated using the non-parametric bootstrap method and a Bayesian inference approach. Uncertainty was then propagated through AP simulations to quantify uncertainty in qNet for each drug. UQ revealed lower uncertainty and more accurate TdP risk stratification by qNet when simulations were run at concentrations below 5× the maximum therapeutic exposure (C_max_). However, when drug effects were extrapolated above 10× C_max_, UQ showed that qNet could no longer clearly separate drugs by TdP risk. This was because for most of the pharmacology data, the amount of current block measured was <60%, preventing reliable estimation of IC_50_-values. The results of this study demonstrate that the accuracy of TdP risk prediction depends both on the intrinsic variability in ion channel pharmacology data as well as on experimental design considerations that preclude an accurate determination of drug IC_50_-values *in vitro*. Thus, we demonstrate that UQ provides valuable information about *in silico* modeling predictions that can inform future proarrhythmic risk evaluation of drugs under the CiPA paradigm.

## Introduction

Drugs that block cardiac ion channels encoded by the *human-ether-à-go-go Related Gene* (hERG) and consequently prolong the QT interval are associated with increased risk of Torsade de Pointes (TdP), a potentially lethal arrhythmia that caused several drugs to be withdrawn from market (Gintant et al., 2016). In 2005, the International Council on Harmonisation (ICH) S7B and E14 guidelines were established to address the issue of TdP liability for new drugs. As stated in these guidelines, their intent was to be used as a screening method to identify drugs that would require more intensive electrocardiographic monitoring of patients in late phase (e.g., phase 3) clinical trials. However, hERG block or QT prolongation does not necessarily correlate with TdP risk, and as a result of these guidelines, many novel compounds are screened out of development because of detected hERG block or QT prolongation without further evaluation of actual TdP risk. Additional insight into TdP risk for hERG-blocking and QT-prolonging drugs can be determined by also assessing whether drugs block inward currents such as, L-type calcium or late sodium (Duff et al., 1987; January and Riddle, 1989; Chézalviel-Guilbert et al., 1995; Guo et al., 2007). The Comprehensive *in vitro* Proarrhythmia Assay (CiPA) is a global initiative to revise the current guidelines with a new set of mechanistic assays that improve the specificity of the proarrhythmia screening process (Fermini et al., 2016).

The CiPA *in silico* assay will test new compounds for the potential to cause TdP by incorporating *in vitro* pharmacology data on multiple ion channels into a mathematical model of the cardiac action potential (AP). The AP model will be used to predict drug effects related to early afterdepolarizations (EADs), which are a known cellular trigger of TdP (Yan et al., 2001). Numerous studies have shown that when outward repolarizing currents such as, I_Kr_ (the current carried by hERG-encoded channels) are blocked in cardiac cells, the resulting imbalance of inward and outward currents prolongs the AP and can, at extreme levels, lead to inward current reactivation and EADs (January and Moscucci, 1992). However, EADs may not occur if a drug also significantly blocks inward currents, leading to a balanced block scenario where the AP is prolonged but inward currents cannot reactivate (Antzelevitch et al., 2004). Because it is difficult to know how much inward vs. outward current block is safe, or how dynamic effects might impact EAD propensity, the purpose of the CiPA *in silico* model will be to assess the integrated effects of multiple ion channel block on TdP risk. As with any model built on inherently variable experimental data, however, confidence in model predictions will depend on the level of uncertainty in model inputs (here, the drug-specific parameters) and the corresponding uncertainty in model outputs (Pathmanathan et al., 2015; Johnstone et al., 2016b). In order for CiPA to provide useful guidance to the drug development and regulatory process, it will be necessary to incorporate uncertainty quantification (UQ) into modeling predictions (Pathmanathan and Gray, 2013; Mirams et al., 2016).

The CiPA *in silico* ventricular AP model and a mechanism-based metric for TdP risk stratification have been trained on a designated set of 12 CiPA compounds with known TdP risk levels (High, Intermediate, or Low, see Table 1). These compounds were selected and categorized by a team of expert clinicians, safety pharmacologists, and electrophysiologists based on adverse event data and published reports (Colatsky et al., 2016). The current CiPA AP model was developed through a series of modifications to the O'Hara-Rudy (ORd) human ventricular AP model (O'Hara et al., 2011). Li et al. (2016) first developed a Markov model of the hERG channel that included temperature-sensitive gating, which was subsequently modified to recapitulate I_Kr_ from the original ORd model, with an added pharamacological component (Li et al., 2017). The hERG/I_Kr_ model was then incorporated into the ORd AP model to produce the I_Kr_-dynamic ORd model. In the CiPAORdv1.0 model, we further optimized the I_Kr_-dynamic ORd model by scaling ionic current conductances to better reflect changes in AP duration observed in human ventricular myocytes when ionic currents were blocked (referred to as the optimized I_Kr_-dynamic ORd model in Dutta et al., 2017). With this model, we derived a new *in silico* biomarker for TdP risk, the qNet metric, which correlated well with *in silico* cell “distance” to EADs and thus provided a continuous marker for EAD susceptibility. Although we showed that the qNet metric could correctly stratify the 12 CiPA training drugs by known TdP risk, uncertainty in these modeling predictions was not evaluated.

**Table 1:** TdP risk levels for the 12 CiPA training compounds.

**Drug**	**CiPA TdP Risk**
Dofetilide	High
Bepridil	High
Sotalol	High
Quinidine	High
Cisapride	Intermediate
Terfenadine	Intermediate
Ondansetron	Intermediate
Chlorpromazine	Intermediate
Verapamil	Low
Ranolazine	Low
Mexiletine	Low
Diltiazem	Low

In this study, methods for applying UQ to the CiPA *in silico* assay are presented. For the 12 CiPA training compounds, we examine the uncertainty in drug-specific kinetics parameters for drug binding and trapping in the I_Kr_-dynamic model. In addition, we examine uncertainty in dose-response curve IC_50_ and Hill coefficients for the remaining six CiPA-selected ionic currents, as this can also be considerable (Elkins et al., 2013). We thereby characterize uncertainty in drug effects on ion channels due to variation in experiments, whatever the cause of this variation may be. We then sample from these probability distributions for the drug effects and run forward simulations to examine the subsequent uncertainty in qNet and TdP risk stratification.

## Materials and methods

### Human ventricular action potential model

The CiPAORdv1.0 model (the optimized model from Dutta et al., 2017) was used for all simulations in this study, in order to evaluate TdP risk for the set of 12 CiPA training compounds listed in Table 1. Parameter values for the model are listed in Tables S1, S2.

### Multiple ion channel pharmacology

Pharmacological effects of the 12 CiPA training compounds on ionic currents were modeled as in Li et al. (2017) and Dutta et al. (2017). The kinetics of hERG block were modeled with the I_Kr_ Markov model from Li et al. (2017), which was fit to voltage clamp data obtained at the U.S. Food and Drug Administration (FDA; parameters listed in Table 2). For six other ionic currents (L-type calcium, I_CaL_; late sodium, I_NaL_; fast sodium, I_Na_; transient outward, I_to_; slowly activating delayed rectifier, I_Ks_; and inward rectifier, I_K1_), drug effects were represented by a simple pore blocking model in which maximal current conductances were reduced according to the Hill equation. Hill equation parameters (Table 3) were fit to data from Crumb et al. (2016). Some of the data have been updated since publication and are available online (see section Software and Data).

**Table 2:** Drug-hERG binding parameters for the 12 CiPA training compounds.

	**log_10_(K_max_)**	**log_10_(EC_50_^n^)**	**log_10_(K_max_/EC_50_n)**
Dofetilide	1.5453 [0.9209, 6.8153]	2.3357 [1.7982, 7.5726]	−0.7905 [−1.0125, −0.6304]
Bepridil	6.7477 [5.4278, 7.1407]	8.1679 [6.803, 8.5243]	−1.4202 [−1.7124, −1.1736]
Sotalol	4.9831 [0.8193, 5.4306]	8.5861 [4.965, 8.9975]	−3.6029 [−4.5017, −3.1522]
Quinidine	2.4404 [1.1871, 6.4189]	4.731 [3.6678, 8.7601]	−2.2906 [−2.7484, −2.0986]
Cisapride	1.0095 [0.839, 1.6553]	1.6265 [1.4211, 2.2125]	−0.6171 [−0.9699, −0.4154]
Terfenadine	5.0095 [1.2953, 6.2265]	5.6123 [1.8881, 6.8917]	−0.6028 [−0.7791, −0.4311]
Ondansetron	5.2355 [1.5791, 6.3269]	7.718 [4.341, 8.7997]	−2.4825 [−2.7992, −2.2702]
Chlorpromazine	5.1984 [4.696, 6.5012]	7.6386 [7.0863, 8.9725]	−2.4402 [−2.7268, −2.2162]
Verapamil	6.2289 [1.5379, 6.803]	8.5258 [4.1385, 8.9922]	−2.2969 [−2.9551, −1.7767]
Ranolazine	1.723 [1.3627, 5.6536]	5.1553 [4.8122, 8.7298]	−3.4324 [−4.0139, −2.9363]
Mexiletine	1.1761 [1.0208, 1.497]	5.8591 [5.3159, 6.5914]	−4.683 [−5.5154, −3.9582]
Diltiazem	5.2613 [1.6549, 5.6663]	8.8246 [5.7087, 8.9997]	−3.5634 [−4.1562, −3.223]
	**n**	**log_10_(K_u_)**	**V_halftrap_**
Dofetilide	1.08 [0.9527, 1.467]	−4.7409 [−4.9767, −4.6633]	−1 [−26.01, −1]
Bepridil	0.9374 [0.8227, 1.074]	−3.7647 [−3.8713, −3.671]	−61.34 [−72.94, −18.36]
Sotalol	0.7513 [0.6594, 0.955]	−1.6527 [−2.0183, −0.4512]	−51.5 [−74.62, −7.756]
Quinidine	0.8488 [0.7775, 1.028]	−2.3869 [−2.3649, −1.7435]	−61.35 [−72.31, −5.445]
Cisapride	0.9615 [0.5928, 1.372]	−3.3808 [−3.4836, −3.2553]	−167.4 [−190.3, −156.5]
Terfenadine	0.6502 [0.5033, 0.7918]	−4.1086 [−4.2938, −4.0023]	−81.63 [−155, −73.87]
Ondansetron	0.891 [0.83, 1.002]	−1.6338 [−1.7335, −1.3971]	−82.2 [−88.69, −77.64]
Chlorpromazine	0.8871 [0.8006, 0.9916]	−1.3306 [−1.7312, −0.7396]	−14.45 [−66.29, −2.865]
Verapamil	1.043 [0.832, 1.317]	−3.088 [−3.1708, −2.6366]	−97.08 [−192, −85.3]
Ranolazine	0.9532 [0.8248, 1.106]	−1.6914 [−1.914, −0.0004]	−94.99 [−176.4, −81.16]
Mexiletine	1.139 [0.956, 1.34]	−1.1479 [−1.4011, −0.016]	−87.51 [−164.8, −77.68]
Diltiazem	0.9382 [0.8612, 1.086]	−0.5498 [−1.0751, 0]	−90.65 [−180.3, −81.18]

*The optimal values are shown with 95% CIs obtained with bootstrapping. Units are as follows: K_max_ (unitless), K_u_ (ms^−1^), EC_50_ (nM), n (unitless), and V_halftrap_ (mV)*.

**Table 3:** Hill equation parameters for the 12 CiPA training compounds.

		**I_CaL_**	**I_K1_**	**I_Ks_**
Dofetilide	pIC_50_	6.5845 [−0.7468, 7.9108]	6.4041 [−0.7384, 7.8736]	N/A
	h	1.163 [0.32, 9.622]	0.765 [0.2685, 9.658]	N/A
Cisapride	pIC_50_	2.0331 [−0.8172, 6.4905]	4.5305 [−0.7411, 6.7042]	1.0906 [−0.767, 6.528]
	h	0.4261 [0.4063, 9.736]	0.5133 [0.2572, 9.63]	0.2921 [0.2409, 9.67]
Bepridil	pIC_50_	5.5516 [5.2752, 5.71]	N/A	4.5432 [3.4422, 4.9682]
	h	0.6486 [0.4351, 0.9191]	N/A	0.7061 [0.3907, 1.142]
Verapamil	pIC_50_	6.6951 [6.6029, 6.7891]	0.4574 [−0.8155, 5.7514]	N/A
	h	1.097 [0.861, 1.43]	0.2728 [0.2526, 9.655]	N/A
Terfenadine	pIC_50_	6.1547 [6.0876, 6.2131]	N/A	3.3982 [0.0077, 5.9477]
	h	0.6601 [0.595, 0.7367]	N/A	0.543 [0.2777, 9.728]
Ranolazine	pIC_50_	N/A	N/A	1.4418 [−0.624, 4.3335]
	h	N/A	N/A	0.5191 [0.3292, 8.066]
Sotalol	pIC_50_	2.1511 [1.7907, 2.3628]	2.5157 [2.385, 2.5955]	2.3745 [2.0951, 2.507]
	h	0.8651 [0.5902, 1.259]	1.204 [0.9066, 1.611]	1.167 [0.7741, 1.698]
Mexiletine	pIC_50_	4.4175 [3.9423, 4.6525]	N/A	N/A
	h	1.031 [0.6484, 1.576]	N/A	N/A
Quinidine	pIC_50_	4.2874 [3.8501, 4.5293]	1.4024 [−0.7594, 5.0793]	5.3099 [5.2008, 5.3813]
	h	0.5892 [0.4384, 0.7362]	0.3468 [0.2715, 9.492]	1.363 [0.9565, 2.122]
Ondansetron	pIC_50_	4.6469 [4.4138, 4.7937]	N/A	3.2443 [2.138, 3.9253]
	h	0.7526 [0.5478, 1.024]	N/A	0.6535 [0.3954, 1.238]
Diltiazem	pIC_50_	6.9504 [6.7786, 7.1267]	N/A	N/A
	h	0.7142 [0.5344, 1.008]	N/A	N/A
Chlorpromazine	pIC_50_	5.0866 [4.9108, 5.2128]	5.0329 [4.8446, 5.1718]	N/A
	h	0.8441 [0.6105, 1.189]	0.6878 [0.5226, 0.8822]	N/A
		**I_to_**	**I_NaL_**	**I_Na_**
Dofetilide	pIC_50_	7.7254 [6.8317, 7.9571]	3.1231 [−0.754, 7.8227]	6.4196 [−0.6142, 8.0307]
	h	0.7712 [0.3735, 1.147]	0.2597 [0.1543, 9.49]	0.892 [0.2235, 9.497]
Cisapride	pIC_50_	3.6594 [−0.6456, 5.6778]	N/A	N/A
	h	0.243 [0.1166, 0.5656]	N/A	N/A
Bepridil	pIC_50_	5.0658 [−0.5052, 5.3383]	5.7414 [5.6743, 5.8074]	5.5333 [5.3948, 5.6158]
	h	3.541 [0.4166, 9.499]	1.416 [1.133, 1.789]	1.164 [0.8083, 1.71]
Verapamil	pIC_50_	4.8719 [1.1464, 5.5056]	5.1532 [−0.6313, 5.8804]	N/A
	h	0.8222 [0.2414, 1.793]	1.031 [0.222, 9.41]	N/A
Terfenadine	pIC_50_	3.6198 [−0.0501, 5.1184]	4.6977 [2.6363, 5.8293]	5.3185 [4.8576, 6.0114]
	h	0.2559 [0.1246, 0.5777]	0.6011 [0.269, 3.232]	1.015 [0.6554, 9.176]
Ranolazine	pIC_50_	N/A	5.1033 [4.9859, 5.2079]	4.1626 [3.2696, 4.5616]
	h	N/A	0.945 [0.7247, 1.256]	1.425 [0.6228, 9.116]
Sotalol	pIC_50_	1.3651 [−0.3529, 2.1817]	N/A	−0.0584 [−0.8951, 2.4926]
	h	0.6632 [0.3213, 1.704]	N/A	0.5089 [0.3913, 8.449]
Mexiletine	pIC_50_	N/A	5.0478 [4.9484, 5.1128]	N/A
	h	N/A	1.409 [1.041, 1.846]	N/A
Quinidine	pIC_50_	5.4575 [5.3999, 5.511]	5.0261 [4.9062, 5.1077]	4.909 [4.6683, 5.0426]
	h	1.282 [1.049, 1.585]	1.337 [1.034, 1.7]	1.494 [1.004, 2.236]
Ondansetron	pIC_50_	2.9901 [−0.8308, 4.4636]	4.7172 [4.6073, 4.8]	4.2391 [3.5217, 4.6469]
	h	0.9891 [0.4407, 9.691]	1.035 [0.8001, 1.399]	1.02 [0.5024, 8.671]
Diltiazem	pIC_50_	−0.4506 [−0.922, 2.6212]	4.6602 [4.5116, 4.7776]	3.9551 [3.2876, 4.8315]
	h	0.1696 [0.1551, 0.364]	0.6779 [0.5485, 0.9082]	0.7022 [0.4484, 9.337]
Chlorpromazine	pIC_50_	1.754 [−0.6914, 4.776]	5.341 [5.2543, 5.4232]	5.3433 [5.221, 5.4298]
	h	0.3654 [0.2318, 8.56]	0.9379 [0.7797, 1.148]	1.995 [1.628, 3.064]

*The optimal fitted values are shown with 95% CIs obtained using Markov-chain Monte Carlo simulation. IC_50_-values are log-transformed as pIC_50_ = −log_10_(IC_50_/c_0_), where c_0_ = 10^9^ nM. Not applicable (N/A) indicates that IC_50_-values were not defined in Li et al. (2017), so the amount of block was assumed to always be 0%*.

### Numerical methods and data analysis

Model equations were written in C and compiled for use with version 3.3 of the R programming language (R Core Team, 2016) and version 1.14 of the deSolve package (Soetaert et al., 2010). Equations were integrated using the lsoda solver with relative and absolute error tolerances of 10^−6^ and other solver settings as default. For computationally intensive bootstrap simulations (see section Drug-hERG Binding Kinetics), a relative tolerance of 10^−3^ was used. Data analysis was performed in R, and figures were produced with version 2.2.0 of the ggplot2 package (Wickham, 2009).

### Simulation protocol for TdP risk evaluation

The CiPAORdv1.0 model was used to simulate APs at a cycle length (CL) of 2 s (stimulus amplitude = −80 μA/μF, duration = 0.5 ms). The model was initialized from control (no drug) steady-state values (Table S3) and paced for 1,000 beats. Drugs were simulated at multiples of their maximum therapeutic concentrations (C_max_, Table S4), ranging from 1 to 10× C_max_ (1× increments) and from 15 to 25× C_max_ (5× increments). At each concentration, TdP risk was evaluated using the metric qNet, defined as the net charge carried by six major currents (I_Kr_, I_CaL_, I_NaL_, I_to_, I_Ks_, and I_K1_) over an entire beat (Dutta et al., 2017). The qNet metric was computed by integrating the sum of the six currents from the start of the stimulus (*t* = 0 s) until the end of the beat (*t* = 2 s) using lsoda (see section Numerical Methods and Data Analysis).

Analysis was performed only on the last 250 beats of the pacing protocol to allow drug effects to reach quasi-steady state for simulations with beat-to-beat instability. Beats in which transmembrane potential (V_m_) failed to depolarize above 0 mV were excluded from analysis, and simulations in which every beat failed to depolarize were excluded from TdP risk evaluation. The maximum slope during repolarization (dV/dt_repol_) was defined as the maximum change in V_m_ (dV/dt) between 30 and 90% repolarization for beats that fully repolarized; as the maximum dV/dt between 30% repolarization and the end of the beat (*t* = 2 s) when V_m_ repolarized by 30% but not 90%; or as the maximum dV/dt between the AP peak and the end of the beat when V_m_ failed to repolarize by 30%. An EAD was defined to have occurred on any beat in which dV/dt_repol_ was greater than zero. Out of the last 250 beats, the beat with the steepest reactivation of the membrane potential (maximum dV/dt_repol_) was used to calculate qNet, whether or not an EAD had occurred.

### Uncertainty characterization

#### Drug-hERG binding kinetics

In Li et al. (2017), time series measurements of the fractional hERG current in the presence of drug were obtained using a modified Milnes voltage clamp protocol (Milnes et al., 2010; Li et al., 2017). Because of the long duration of the protocol, each cell could only be tested at a single drug concentration, and the drug-hERG binding and trapping parameters (see Table 2) were fit to the fractional current traces measured during a voltage step to 0 mV, averaged across cells by concentration. Specifically, each dataset *y* consisted of a set of fractional current time series observations *x*_*c,i*_(*t*) (*c* = 1, 2, …, *m*, where *m* is number of the concentrations tested; *i* = 1, 2, …, *n*_*c*_, where *n*_*c*_ is the number of cells tested at the *c*th concentration; and *x*_*c,i*_(*t*_*j*_) were independent between concentrations). The mean drug response at the *c*th concentration was ${\bar{x}}_{c}\left(t\right)=\frac{1}{n_{c}}\overset{n_{c}}{\underset{i=1}{\sum }}x_{c,i}\left(t\right)$ (i.e., the average of fractional current traces across cells), and the overall mean response $\bar{y}=\left({\bar{x}}_{1}\left(t\right),{\bar{x}}_{2}\left(t\right),\ldots ,{\bar{x}}_{m}\left(t\right)\right)$ (i.e., the set of average fractional current traces at each concentration) was used to fit the optimal drug-hERG kinetics parameters ($\hat{\theta }(\bar{y})$). Parameters were fitted using the Covariance Matrix Adaptation Evolutionary Strategy (CMA-ES) (Hansen, 2006), with version 1.0-11 of the cmaes package (Trautmann et al., 2011). Details on the CMA-ES implementation can be found in the Supplemental Methods. Bounds for the dynamic drug-hERG binding parameters used to fit bootstrap samples can be found in Table S5.

The non-parametric bootstrap method was used to characterize uncertainty in the fitted parameters (Efron and Tibshirani, 1986). Observations $x_{c,i}^{*}$(t) were randomly drawn with replacement from *x*_*c,i*_(*t*) to obtain a bootstrap sample $y_{b}^{*}$ of the same size as the original dataset, with an identical number of observations per concentration. A total of 2,000 bootstrap samples (*b* = 1, 2, …, 2000) were generated using version 1.3-18 of the boot package (Davison and Hinkley, 1997; Canty and Ripley, 2016). The mean response ${\bar{y}}_{b}^{*}$ for each bootstrap sample was then computed in the same manner as ȳ and used to refit the drug-hERG kinetics parameters ($\hat{\theta }({\bar{y}}_{b}^{*})$), yielding a joint sampling distribution of drug-hERG parameters.

#### Dose-response curves

For other ionic currents, uncertainty in dose-response curves was characterized using a Bayesian inference approach. Version 1.3.5 of the FME package was used to fit Hill equation parameters and to characterize uncertainty, using Markov-chain Monte Carlo (MCMC) simulation with the delayed rejection and adaptive Metropolis algorithm (Soetaert and Petzoldt, 2010). The percentage of ionic current block was assumed to be a normal random variable located at the Hill equation response curve with unknown variance σ^2^. Log-transformed IC_50_-values [pIC_50_ = −log_10_(IC_50_/c_0_), where c_0_ = 10^9^ nM] were bounded to the range [−1, 19] for fitting and MCMC simulation (bounding IC_50_-values between 10^−10^ and 10^10^ nM). Hill coefficients (h) were bounded to the range [0, 10]. Optimal IC_50_ and Hill coefficient (h)-values were fit using non-linear least squares (see Table 3). The joint probability distribution of IC_50_ and h was estimated using MCMC simulation. A uniform prior distribution was used for pIC_50_ and h. The error variance σ^2^ was considered a nuisance parameter and was sampled as conjugate priors from an inverse gamma distribution during MCMC simulation. The proposal distribution was multivariate normal. A total of 2,000 MCMC samples (pIC_50_, h) were saved for each drug-current combination to form a joint sampling distribution of Hill equation parameters (see Supplemental Methods for implementation details).

#### Credible intervals

Variability of model inputs (parameters) or outputs (predicted responses) was summarized with 95% credible intervals (95% CIs, the 2.5–97.5% quantiles of the marginal distributions).

### Uncertainty propagation

Samples from the joint distribution of drug-hERG parameters and the joint distributions of Hill equation parameters for a particular drug were assumed to be independent and were combined in AP simulations to assess the uncertainty in qNet (see section Simulation Protocol for TdP Risk Evaluation). One sample from each distribution was selected in sequential order (e.g., the first sample from each distribution) to form a set of parameters that defined a single sample from the drug-effect probability distribution. This was repeated until all parameter samples were exhausted, generating a sampling distribution of 2,000 drug-effect samples per drug (referred to as uncertainty inputs), which. Each input was simulated with the CiPAORdv1.0 model to assess variability in AP model outputs (qNet, see section Simulation Protocol for TdP Risk Evaluation). Variability in qNet was quantified with 95% CIs. Sampling distributions of qNet were visualized with violin plots.

### Cross validation

Leave-one-out cross validation (LOOCV) (Hastie et al., 2009) was used to assess the accuracy of TdP risk stratification at each simulated concentration relative to C_max_. The CiPA Low, Intermediate, and High TdP risk levels (Table 1) were given numerical category values of 0, 1, and 2, respectively. At each concentration (1−25× C_max_), a classifier was trained on all samples from the qNet distributions of all but one of the training drugs. The classifier was based on proportional odds logistic regression using the lrm function from version 4.5-0 of the rms package (Harrell, 2016). The numerical tolerance was set to 10^−10^ and the maximum number of iterations was set to 10^6^ for fitting. Each sample of the remaining, “left out” drug was then assigned to the category with the highest probability based on logistic regression results. The predicted probability of each category [P(x), where x is 0, 1, or 2] for the “left out” drug was computed as the fraction of samples assigned to that category, and the prediction error for that drug was computed as the mean absolute difference between the assigned and actual TdP category over all samples. This procedure was repeated for all 12 training drugs, and the mean and standard deviation of prediction errors at each concentration were computed to evaluate overall TdP risk stratification performance.

### Software and data

The software and data used in this study are available at https://github.com/FDA/CiPA.

## Results

### Uncertainty in drug-hERG binding kinetics

**Figure 1:**
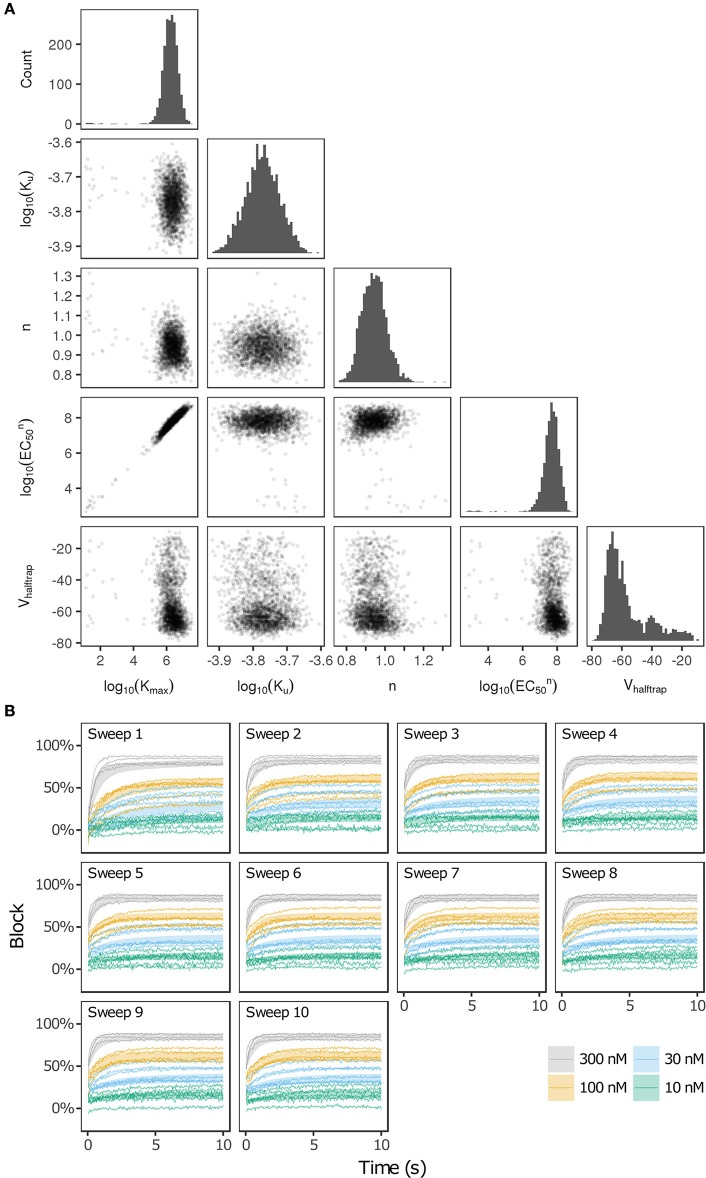
Uncertainty in bepridil-hERG binding kinetics. **(A)** The joint probability distribution of K_max_ (maximum drug effect at saturating concentrations), K_u_ (rate of drug unbinding), *n* (Hill coefficient of drug binding), EC_50_^n^ (nth power of the half-maximal drug concentration), and V_halftrap_ (drug trapping potential) was estimated by bootstrapping. Plots on the diagonal show the marginal histograms of each parameter (log-transformed in some cases). Plots below the diagonal show pairwise scatter plots of the fitted parameters for 2,000 bootstrap samples. **(B)** Kinetics of hERG block during 10 sweeps of a modified Milnes voltage-clamp protocol (Milnes et al., 2010; Li et al., 2017). Shaded areas show the range of block produced by the parameters from **(A)**. Lines show the experimental results used to fit the data (down-sampled 5× for clarity).

Bootstrapping was performed with voltage clamp data from Li et al. (2017) in order to estimate the joint probability distribution of fitted drug-hERG dynamic binding parameters. The 95% CIs of hERG binding parameters for the 12 CiPA training drugs (Table 1) are listed in Table 2. Parameter fitting results for bepridil are illustrated in Figure 1A. The rate of bepridil unbinding (K_u_) had a relatively narrow 95% CI [10^−3.8713^, 10^−3.671^ ms^−1^], indicating that this parameter was well-constrained by the experimental data and uncertainty in its value was low. In contrast, the pairwise scatter plot of log_10_(K_max_) and log_10_(EC_50_^n^) revealed a strong correlation between the two parameters, and their fitted ranges spanned several orders of magnitude. The pairwise scatter plots for other training drugs displayed similar correlations between log_10_(K_max_) and log_10_(EC_50_^n^) (panel A in Figures S1–S11).

The large uncertainty in K_max_ and EC_50_^n^ did not produce a similar degree of variability in the kinetics of hERG block, however. In Figure 1B and panel B of Figures S1–S11, shaded areas indicate the 95% CI of the block predicted by parameters in Figure 1A and panel A of Figures S1–S11. The variability in hERG block was much more limited than the variability in K_max_ or EC_50_^n^, which was not surprising because Li et al. (2017) showed that for most of the 12 training drugs, there was a near-linear relationship between drug concentration and binding rate, occurring when the fitted EC_50_-value was much greater than the maximum drug concentration tested. For example, the optimal EC_50_-value of bepridil was 10^8.7^ nM, and the bootstrap-estimated 95% CI was [10^7.0^, 10^9.7^], but the maximum bepridil concentration tested was 300 nM, or roughly 10^2.5^ nM. In such cases, the E_max_ equation defining the sigmoidal dose-response relationship of drug binding [E_max_ = K_50_^n^))] was linearly approximated by E_max_≈(K_max_/EC_50_^n^)^*^D^n^, and the ratio K_max_/EC_50_^n^ effectively becomes a single identifiable parameter. Thus, the 95% CIs for log_10_(K_max_/EC_50_^n^) were much narrower than the 95% CIs for log_10_(K_max_) and log_10_(EC_50_^n^) (Table 2). The E_max_ equation was chosen to model drug binding because of its flexibility in accommodating both linear and sigmoidal dose-response relationships. As a result, for those compounds whose drug binding mode is actually linear, the ratio but not the individual values of the two correlated parameters were identifiable (Li et al., 2017).

**Figure 2:**
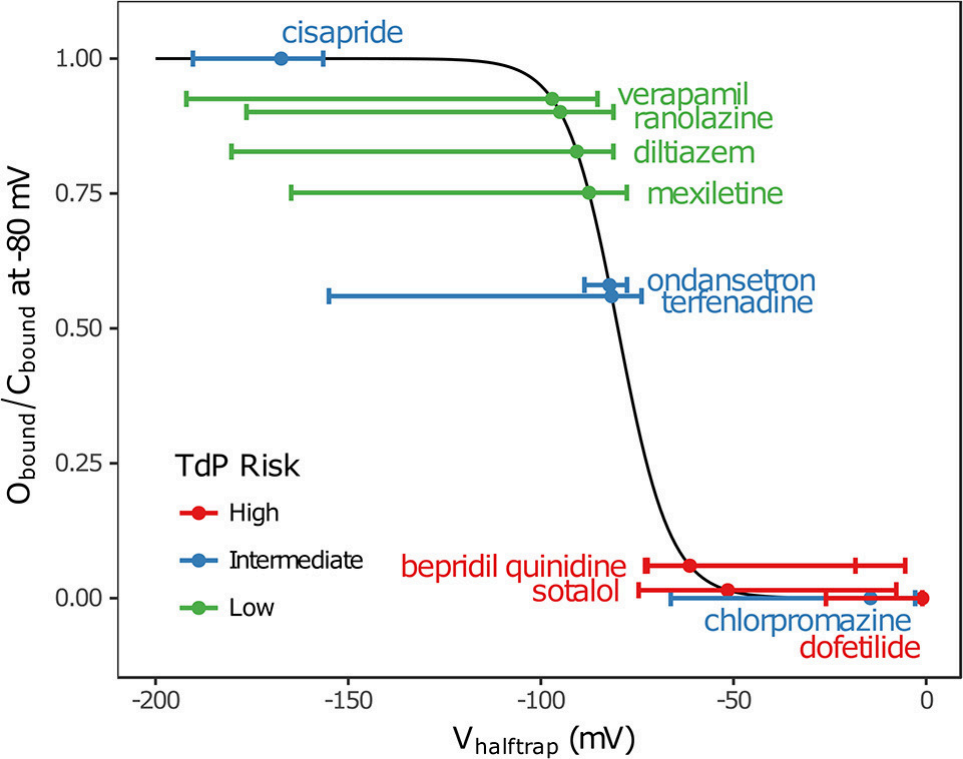
Uncertainty in drug trapping for the 12 CiPA training drugs. Fitted V_halftrap_-values (points) are plotted along the curve defining the resulting steady-state fraction of open-bound to close-bound channels (O_bound_/C_bound_) at V_m_ = −80 mV. The 95% CIs (horizontal error bars) were estimated with bootstrapping. High TdP-risk drugs are in red, Intermediate-risk drugs are in blue, and Low-risk drugs are in green. Intermediate-risk drugs were indistinguishable from Low- and High-risk drugs.

In addition, multimodality (the presence of multiple peaks in the sampling distribution) was frequently observed in other hERG kinetics parameters (Figures S1–S11), in particular with V_halftrap_. In the hERG binding model, V_halftrap_ is a drug trapping parameter that determines the steady-state fraction of open-bound (untrapped) to close-bound (trapped) channels. Li et al. (2017) demonstrated that the High- and Low-risk CiPA training drugs could be separated by this single parameter (V_halftrap_ > −65 mV for High-risk drugs, while V_halftrap_ < −85 mV for Low-risk drugs). The multimodality identified in V_halftrap_ sampling distributions raised the question of whether this trend still holds under uncertainty analysis. As shown in Figure 2, the 95% CIs of V_halftrap_ were quite wide for most drugs, but much of this variability covered ranges where the ratio of open- to close-bound channels (O_bound_/C_bound_) at −80 mV was relatively flat, near 1 for Low-risk drugs (green bars) or near 0 for High-risk drugs (red bars). In the steepest region of the O_bound_/C_bound_ curve, V_halftrap_ distributions of High- vs. Low-risk drugs were well-separated (upper credible bounds < −77 mV for Low-risk drugs, lower credible bounds > −75 mV for High-risk drugs). Thus, UQ identified consistently low or high levels of trapping for Low- vs. High-risk drugs, respectively, providing increased confidence in the V_halftrap_ trend identified by Li et al. (2017). Note that with or without UQ, the V_halftrap_-values of Intermediate-risk drugs (blue bars and points) other than chlorpromazine were generally indistinguishable from Low-risk drugs, and chlorpromazine was indistinguishable from High-risk drugs, indicating that the degree of drug trapping is not sufficient to stratify compounds into the three CiPA risk levels.

### Uncertainty in dose-response curves

**Figure 3:**
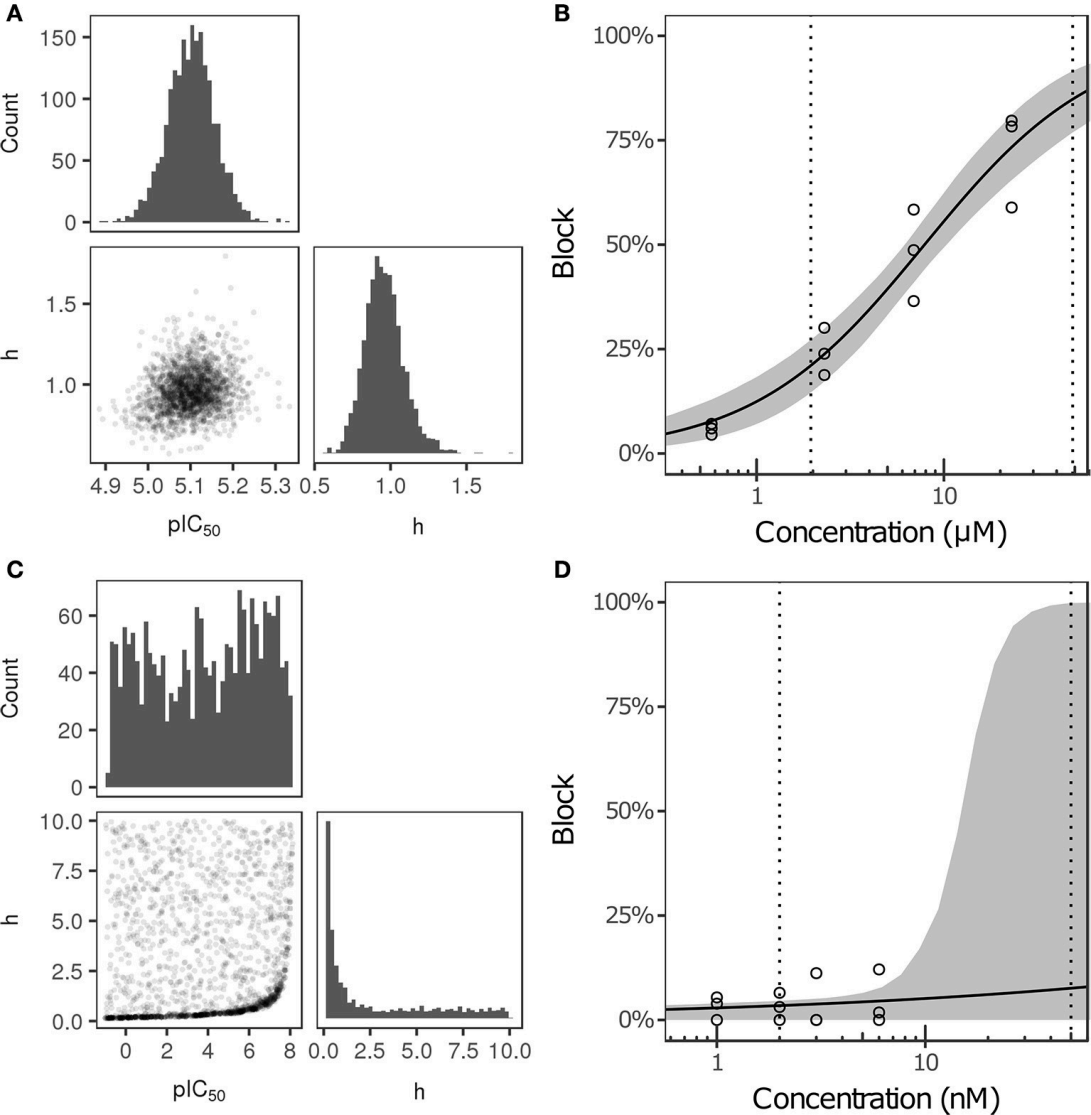
Uncertainty in the dose-response relationship of late sodium current (I_NaL_) block by ranolazine **(A,B)** and dofetilide **(C,D)**. **(A,C)** show the joint distribution of pIC_50_ and Hill coefficient (h)-values, estimated with a Bayesian inference approach. Marginal histograms are displayed on the diagonal plots, and pairwise scatter plots are below the diagonal (2,000 samples per drug). IC_50_-values are in nM. **(B,D)** show the dose-response relationships for the two drugs. Solid lines show the Hill equation defined by IC_50_- and h-values from Li et al. (2017). Shaded areas denote the 95% CI of the percentage block at each concentration, as determined by the parameters in **(A,C)**. Circles are the experimental values used to fit the dose-response curves. Vertical dotted lines indicate the limits of the concentration range used in AP simulations (1−25× C_max_).

Bayesian inference was used to estimate the joint probability distribution of Hill equation parameters characterizing steady-state I_Na_, I_CaL_, I_NaL_, I_to_, I_Ks_, and I_K1_ block by each of the 12 CiPA training drugs. MCMC simulation was not performed for drug-current combinations that did not have defined IC_50_-values in Li et al. (2017), which were assumed to have 0% block. Parameter fitting results are summarized in Table 3. Some MCMC simulations produced joint sampling distributions with a single well-defined peak, such as, that of ranolazine-I_NaL_ (Figure 3A). The mean parameter values of this distribution (pIC_50_ = 5.0958, h = 0.9594) were close to the optimal fitted values (pIC_50_ = 5.1033, h = 0.945), and the 95% CIs [pIC_50_ (4.9859, 5.2079), h (0.7247, 1.256)] were relatively narrow, indicating that uncertainty in these parameters was low. Consequently, the variability in dose-response curves defined by these parameters was also low. At any given concentration, uncertainty in ranolazine-I_NaL_ block (i.e., the width of its 95% CI) was <16% (Figure 3B, shaded area), reflecting the variability observed in experiments (circles). Note that uncertainty in ranolazine- I_NaL_ block did not increase at concentrations beyond the highest tested (23 μM) because the well-constrained dose-response curve allowed for extrapolation beyond experimentally tested concentrations.

For other MCMC simulations such as, dofetilide-I_NaL_, an inverse relationship of possible IC_50_- and h-values was observed, without a defined peak (Figure 3C). Furthermore, many MCMC samples reached near the bounds imposed on IC_50_ and h during fitting [95% CIs for pIC_50_ (−0.754, 7.8227] and h [0.1543, 9.49)]. This was symptomatic of having insufficient experimental data to constrain IC_50_-values, as the maximum measured I_NaL_ block was 12.1% at 3× C_max_, the highest concentration tested (Figure 3D, circles). Although an optimal fit could be defined using least squares (solid line), confidence in the fitted parameters was low, and uncertainty in predicted block increased abruptly above 3× C_max_. At 10× and 25× C_max_, the 95% CIs of predicted block were [0, 82.8%] and [0, 99.8%], respectively, reaching close to the maximum possible range (shaded area). Thus, under circumstances where insufficient current block was achieved in experiments, uncertainty in the dose-response relationship became very high when extrapolating beyond the tested concentrations. Similar findings were obtained with other drug-current combinations (Table 3 and Figures S12–S62).

**Figure 4:**
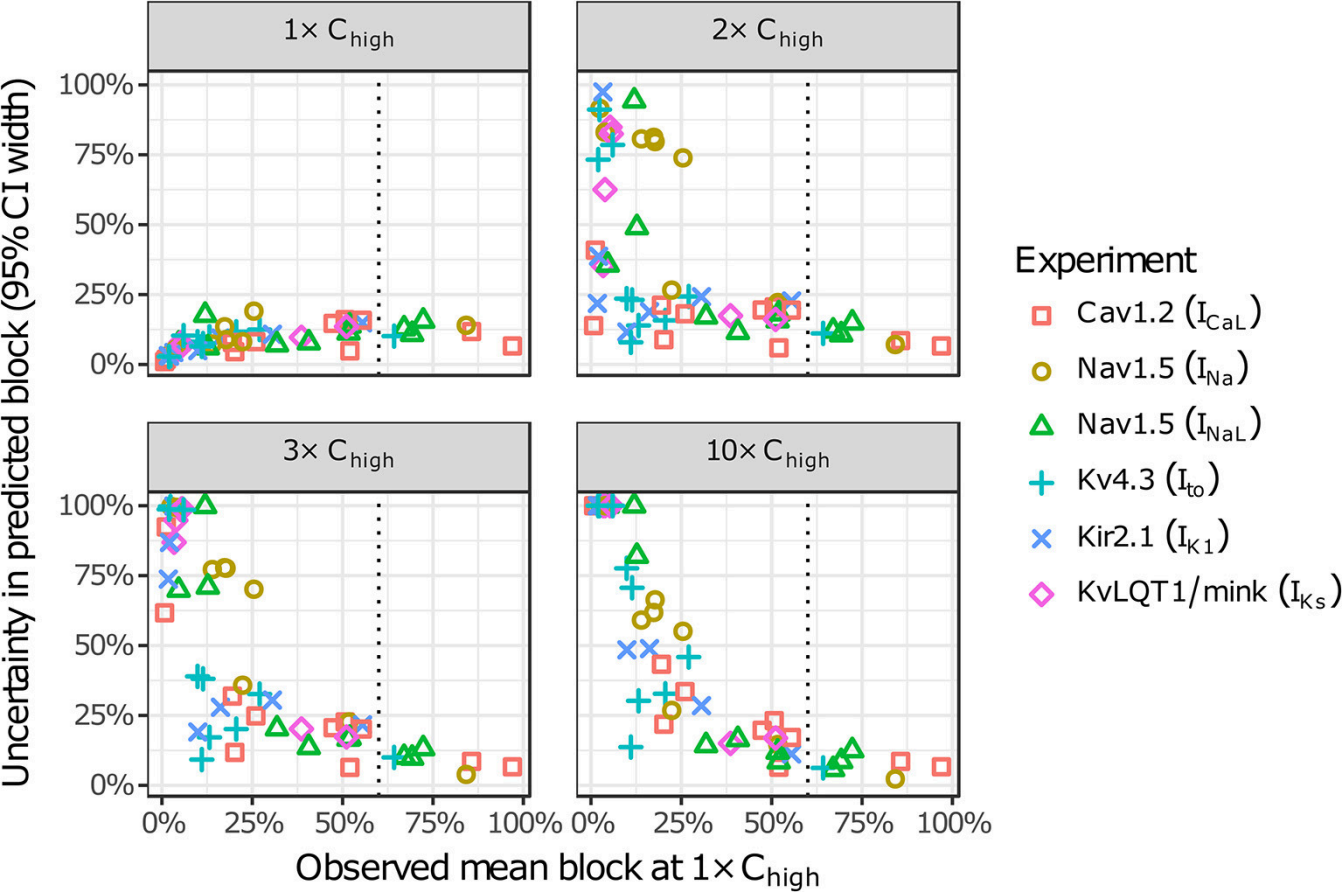
Uncertainty in dose-response curves at extrapolated drug concentrations. Current block experiments were performed for six ionic currents (see legend) with the 12 CiPA training drugs (72 drug-current combinations total with 19 excluded, see Table 3). Dose-response curves were fitted for each experiment and extrapolated above the highest experimentally tested drug concentration (C_high_). Uncertainty in dose-response curves was quantified at 1×, 2×, 3×, and 10× C_high_ as the width of the 95% CI for the predicted percentage block, plotted as a function of the mean experimentally observed block at 1× C_high_. Vertical dotted line is drawn at 60% observed mean block, denoting an approximate lower limit on the mean block that was observed at 1× C_high_ in experiments for which uncertainty remained low (<16%) at higher concentrations.

The amount of uncertainty in predicted block (measured as the width of the 95% CI) was examined as a function of the mean block achieved at the highest tested concentration (C_high_). Table 4 lists the mean block measured in experiments at 1× C_high_ for the 12 CiPA training drugs (some drugs had a different C_high_ for different channels). The resulting uncertainty in the amount of drug block at concentrations above C_high_ is depicted in Figure 4. At 1× C_high_, uncertainty was <25% for all drug-current combinations, indicating that variability in the experimental observations was low. When uncertainty was quantified at extrapolated concentrations (2×, 3×, and 10× C_high_), differences were observed between experiments with low and high amounts of block at 1× C_high_. When <30% mean block was measured at 1× C_high_, uncertainty was >25% for most dose-response curves and reached close to 100% in several cases. But when >60% mean block was measured at 1× C_high_, uncertainty at the extrapolated concentrations was <16%. Thus, UQ results for this dataset suggest that >60% block should be achieved experimentally if dose-response curves are to predict drug effects beyond the tested concentrations. Although >60% block was achieved in hERG experiments with the 12 CiPA training drugs, none of the training drugs were tested at concentrations producing >60% block for all six non-hERG ionic currents (which would be unlikely other than for quinidine, given the selectivity of these compounds). This analysis therefore suggested that drug effects could only be reliably predicted at the highest experimentally tested concentration for which data on all six non-hERG ionic currents were available (Table 4).

**Table 4:** Mean current block at the highest drug concentrations tested in experiments (C_high_).

**Drug**	**C_high_**	**× C_max_**	**I_CaL_ (%)**	**I_K1_ (%)**	**I_Ks_ (%)**	**I_Na_ (%)**	**I_NaL_ (%)**	**I_to_ (%)**
Dofetilide	6 nM	3	1.2	3.4	0.2	2.5	4.6	27.0
Bepridil	3 uM	90.9	50.7	0.5	16.2	51.7	67.0	2.4
Sotalol	2100 uM	143.0	26.0	38.6	30.6	3.9	11.0	11.4
Quinidine	5.4 uM	1.7	20.2	5.6	55.5	22.3	31.8	64.2
Cisapride	125 nM	48.1	0.7	5.3	1.8	2.4	0.0	13.2
Terfenadine	800 nM	200	52.0	0.0	3.3	14.0	12.7	20.6
Ondansetron	20 uM	143.9	47.4	3.0	9.9	25.5	51.6	2.0
Chlorpromazine	10.5 uM	276.3	55.4	51.1	5.2	84.2	69.2	6.0
Verapamil	1 uM	12.3	85.7	3.9	–	0.5	12.0	9.9
	500 nM	6.2	–	–	2.4	–	–	–
Ranolazine	23 uM	11.8	2.5	0.3	2.1	17.4	72.3	–
	69 uM	35.4	–	–	–	–	–	26.5
Mexiletine	10 uM	2.4	19.5	0.6	0.0	6.1	51.9	1.0
Diltiazem	12.5 uM	102.5	97.0	4.6	0.0	17.7	40.6	11.0

*Concentrations are also expressed as multiples of the maximum therapeutic concentration (× C_max_). Because some ionic current experiments used different test concentrations, verapamil and ranolazine both have two entries in the table*.

### Propagation of uncertainty to AP simulations

**Figure 5:**
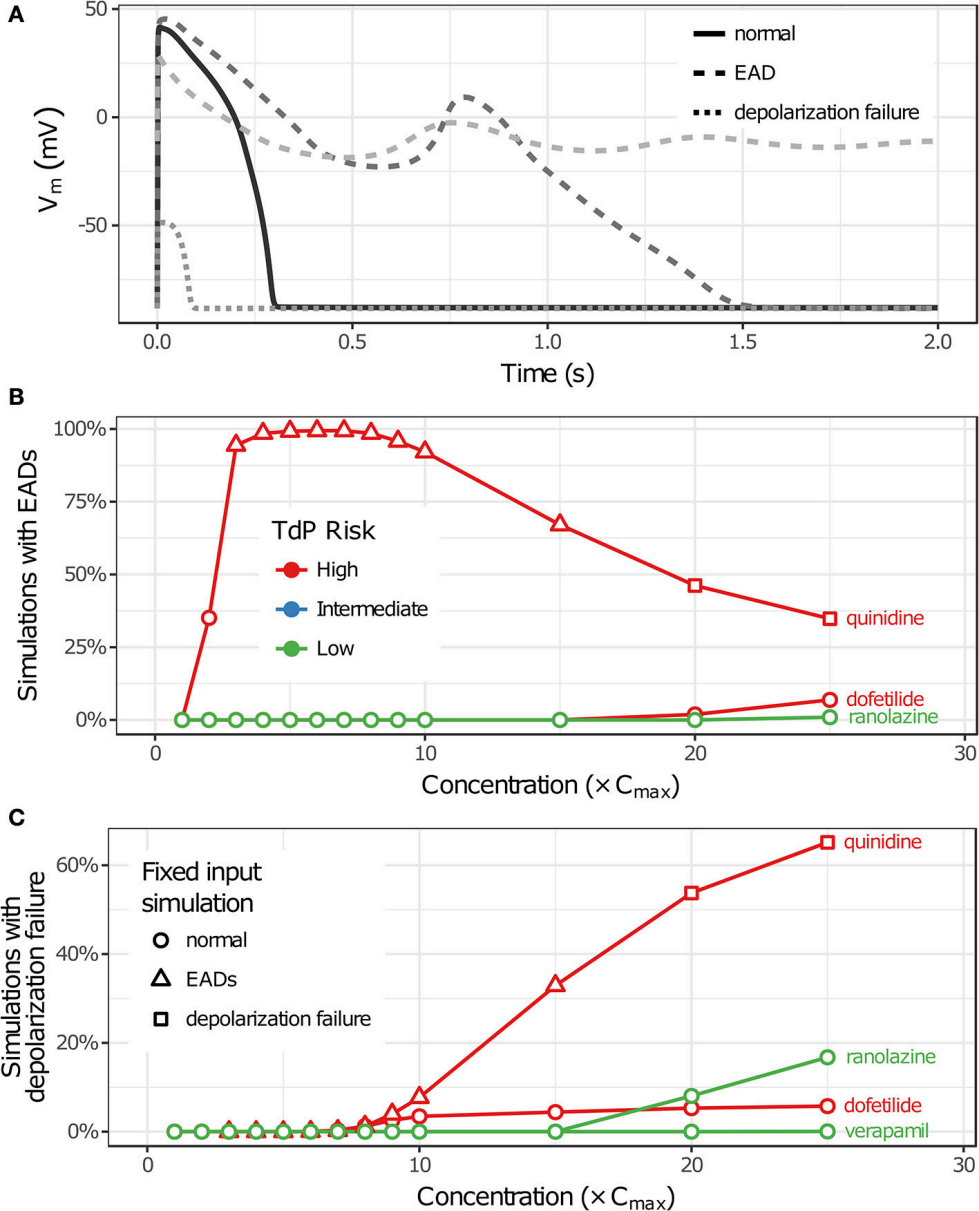
Repolarization and depolarization abnormalities in AP simulations. **(A)** Traces showing representative examples of beats with normal APs (solid), EADs (dashed), or depolarization failure (dotted). **(B,C)** The percentage of uncertainty-input simulations (2,000 total) in which EADs occurred **(B)** or which had complete depolarization failure **(C)** is shown as a function of drug concentration in **(B,C)**, respectively. Only results for drugs that had these events at the simulated concentrations (1−25× C_max_) are plotted. (Note that ranolazine had 19 simulations with EADs at 25× C_max_; verapamil only had one instance of depolarization failure occurring at 25× C_max_.) Markers indicate whether simulations with fixed inputs produced normal Aps (circles), EADs (triangles), or depolarization failure (squares).

Uncertainty in drug-hERG kinetics and dose-response curves was propagated to AP simulations to explore its impact on TdP risk stratification for the 12 CiPA training drugs. For each drug, the optimal drug-hERG parameters and Hill equation parameters (referred to as fixed inputs) were used to simulate APs, as in previous studies (Dutta et al., 2017; Li et al., 2017). In addition, a total of 2,000 drug-effect uncertainty samples per drug (referred to as uncertainty inputs) were simulated in order to estimate the distribution of drug effects derived from uncertainty characterization (see section Uncertainty in Drug-hERG Binding Kinetics–Uncertainty in Dose-Response Curves). Individual beats were classified as having normal APs, EADs, or depolarization failure (Figure 5A), and each simulation was classified as having EADs, complete depolarization failure, or normal otherwise (see section Simulation Protocol for TdP Risk Evaluation). As drug concentration increased from 1 to 25× C_max_ in uncertainty-input simulations, repolarization and depolarization abnormalities became more frequent for some training drugs. EADs occurred in quinidine, dofetilide, and ranolazine simulations (Figure 5B), and depolarization failure occurred in quinidine, dofetilide, ranolazine, and verapamil simulations (Figure 5C). However, the frequency of these events was generally low except in quinidine simulations, which had EADs in >90% of simulations at 3–10× C_max_ and depolarization failure in >50% of simulations at ≥20× C_max_. While EADs are mechanistically linked to TdP, depolarization failure constitutes a different type of rhythm disturbance; therefore, simulations with depolarization failure were removed from further analysis. The remaining simulations represented the conditional distribution of drug effects, given that depolarization failure did not occur at a particular concentration.

### Impact of uncertainty on TdP risk stratification

**Figure 6:**
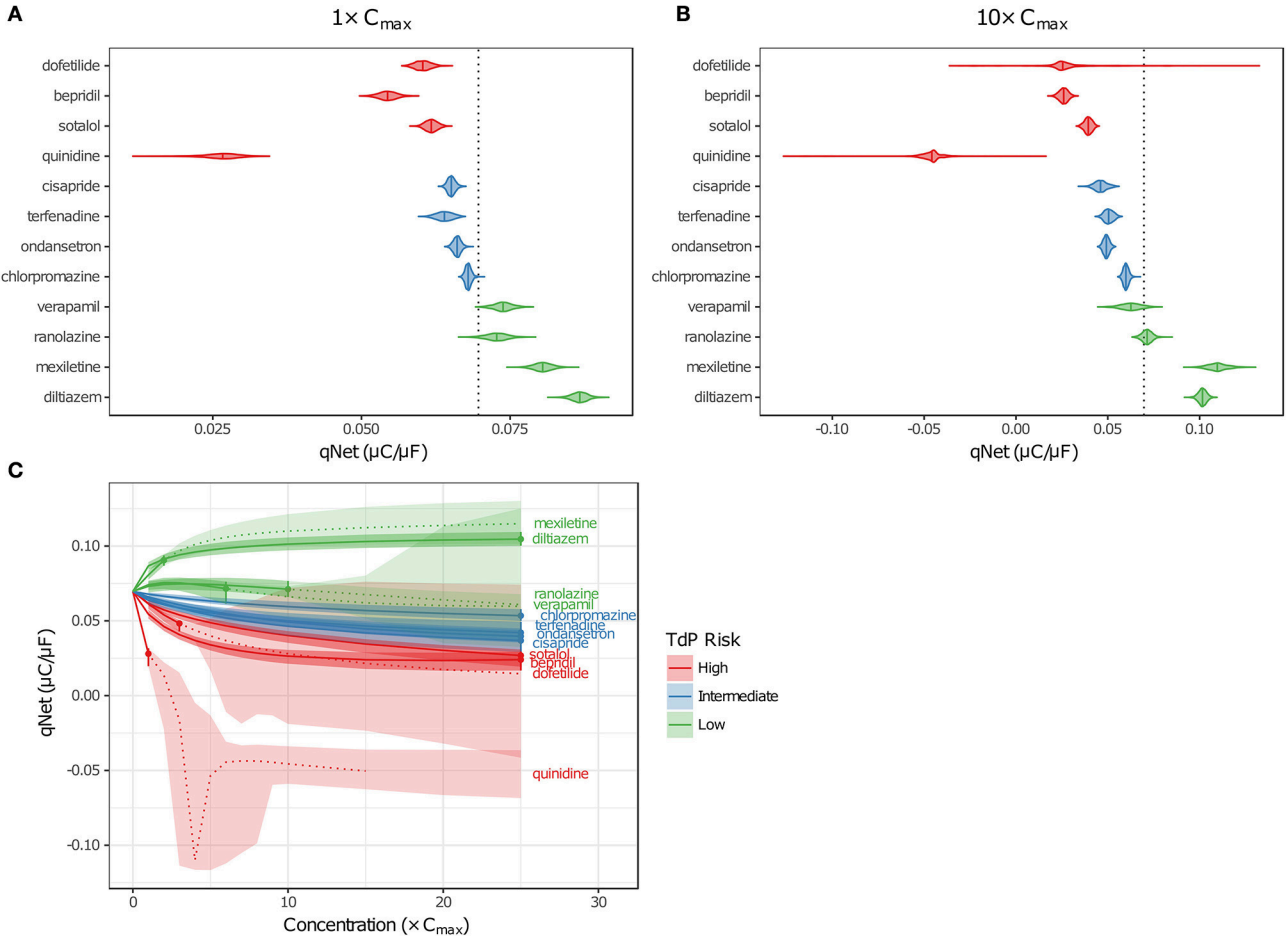
Uncertainty in qNet for the 12 CiPA training drugs. Violin plots are shown for qNet distributions at 1× **(A)** and 10× **(B)** C_max_, based on uncertainty-input simulations. Dotted line indicates the control (no drug) value of qNet. **(C)** qNet at 1−10× C_max_ (1× increments) and 15−25× C_max_ (5× increments). Shaded areas indicate the 95% CIs of qNet obtained from uncertainty-input simulations. Points indicate the highest simulated concentration for which complete experimental data on six non-hERG currents were available. Fixed-input results are shown below (solid lines) or above (dotted lines) this concentration. Likewise, uncertainty-input results are indicated below (dark shaded areas) or above (light shaded areas) this concentration. Simulations with depolarization failure (Figure 5B) were excluded from the results. For all panels, High TdP-risk drugs are in red, Intermediate-risk drugs are in blue, and Low-risk drugs are in green.

Although EADs are a mechanistic marker for TdP risk, stratification based on EADs was not possible because they occurred very rarely in simulations, and not at all for many High Risk compounds at free C_max_. Instead, Dutta et al. (2017) proposed to use the *in silico* metric qNet (the net charge carried by major AP currents during one paced beat at steady state) as an indicator of how far a cell is at a particular drug concentration from producing an EAD. The qNet metric was used in the present study as a marker of TdP risk because it successfully stratified the 12 CiPA training drugs at a range of concentrations in the previous study by Dutta et al. (2017). The calculation of qNet was updated to include simulations in which EADs occurred (see section Simulation Protocol for TdP Risk Evaluation) so that the sampling distributions of qNet would accurately reflect the uncertainty in drug parameters (excluding those that produced depolarization failure). As expected, the values of qNet obtained with uncertainty-input simulations trended according to TdP risk (Figures 6A,B). At a given concentration, median qNet-values decreased between the Low, Intermediate, and High TdP-risk drugs, indicating that outward currents were diminished and inward currents became increasingly dominant at higher risk levels. Note also that extreme negative values of qNet occurred when EADs were present (Figure 6B), reflecting the higher TdP risk evident in these simulations.

Variability in qNet increased as uncertainty in drug effects increased. At 1× C_max_, the distribution of qNet-values for each drug was relatively narrow, and as a result, only a small amount of overlap was observed between adjacent TdP risk levels (Figure 6A). At 10× C_max_, however, the distribution of qNet-values for dofetilide (a High-risk drug) contained several outliers, which encompassed the values for all other drugs except the most negative quinidine values (Figure 6B). These outliers resulted from the high degree of uncertainty in dose-response curves for dofetilide above the highest concentration tested (3× C_max_), particularly with inward currents. As discussed in section Uncertainty in Dose-Response Curves, uncertainty in I_NaL_ block by dofetilide increased dramatically above 3× C_max_ (Figure 3D, shaded area). A similar pattern occurred for I_CaL_ block by dofetilide (Figure S12), with high uncertainty in predicted block at 10× C_max_ [95% CI (0%, 97.6%)]. Because qNet reflects the balance of inward currents (I_NaL_ and I_CaL_) and outward currents (mainly I_Kr_), the effects of I_Kr_ block by dofetilide were offset in simulations with significant block of I_NaL_ or I_CaL_, resulting in the “safe” outliers for dofetilide at 10× C_max_ with very high qNet-values. On the other hand, simulations with very little I_NaL_ or I_CaL_ block led to “dangerous” outliers with very low or negative qNet-values.

Poor separation of qNet between TdP risk levels was apparent at higher drug concentrations, due primarily to the increased uncertainty in drug effects. Dutta et al. (2017) showed that with fixed model simulations, perfect separation in qNet occurred for the 12 CiPA training drugs at 1–25× C_max_. However, our analysis of dose-response uncertainty in section Uncertainty in Dose-Response Curves suggests that qNet may be highly variable above experimentally tested concentrations. In Figure 6C, fixed-input simulation results are shown for concentrations up to (solid lines) and including (point) the maximum simulated concentrations for which complete drug block data on all six non-hERG ionic currents was available; above these concentrations, fixed-input results are plotted as dotted lines. At 1× C_max_, data were available for all 12 CiPA training drugs. Above 1× C_max_, however, some data were unavailable for quinidine (>1.7× C_max_), mexiletine (>2.4× C_max_), dofetilide (>3× C_max_), verapamil (>6.2× C_max_), and ranolazine (>11.8× C_max_; see Table 4). Nevertheless, near 1× C_max_, the 95% CIs of qNet remained largely separated between TdP risk levels, indicating that uncertainty at these concentrations was low enough to stratify the training drugs (shaded areas). At >4× C_max_, however, overlap between different risk levels increased due to the higher variability in qNet sampling distributions, particularly for verapamil and dofetilide. However, increased uncertainty in qNet was not the sole factor affecting TdP risk separation. The qNet-values for verapamil and ranolazine (Low-risk drugs) also drifted closer to those of chlorpromazine (Intermediate-risk) at >4× C_max_, further increasing the overlap between these risk levels, though qNet-values for fixed-input results remained separate.

The accuracy of TdP risk stratification as a function of concentration was assessed using LOOCV. At each concentration relative to C_max_, a classifier was trained on qNet uncertainty samples for 11 of the 12 training drugs and then used to predict the probabilities of each TdP risk level for the remaining drug (see section Cross Validation). At 1× C_max_, the maximum probability always occurred at the correct TdP risk level, but several drugs had non-zero probabilities for the incorrect TdP risk level (Table 5). In contrast, when LOOCV was performed at 1× C_max_ in Dutta et al. (2017), two drugs (terfenadine and chlorpromazine) were misclassified on the basis of fixed-input results (equivalent to a predicted 100% probability of the drug being in the wrong category). As a result, although LOOCV prediction errors were non-zero for more drugs when uncertainty was considered, the overall mean prediction error was lower as compared to fixed-input results (0.09 vs. 0.17). At 10× C_max_, however, mean prediction error was higher when the classifier was trained on uncertainty-input results rather than fixed-input results (0.23 vs. 0.08) because of increased prediction errors for dofetilide, sotalol, cisapride, and verapamil. This was due to the low level of block achieved experimentally for many non-hERG currents, which led to high uncertainty in qNet when drug effects were extrapolated above the tested concentrations. Thus, uncertainty analysis produced more robust TdP risk predictions near concentrations with experimental data for all currents but less robust predictions at concentrations for which extrapolation of drug effects was unreliable due to insufficient levels of block (<60%) measured experimentally.

**Table 5:** Leave-one-out cross validation for TdP risk prediction at 1× C_max_.

	**Left-out drug**	**Category**	**P(0)**	**P(1)**	**P(2)**	**Prediction error**
1× C_max_	Dofetilide	2	0	0.033 (0)	0.967 (1)	0.033 (0)
	Bepridil	2	0	0	1	0
	Sotalol	2	0	0.3475 (0)	0.6525 (1)	0.3475 (0)
	Quinidine	2	0	0	1	0
	Cisapride	1	0	1	0	0
	Terfenadine	1	0	0.5455 (0)	0.4545 (1)	0.4545 (1)
	Ondansetron	1	0	1	0	0
	Chlorpromazine	1	0.1575 (1)	0.8425 (0)	0	0.1575 (1)
	Verapamil	0	0.9995 (1)	0.0005 (0)	0	0.0005 (0)
	Ranolazine	0	0.9215 (1)	0.0785 (0)	0	0.0785 (0)
	Mexiletine	0	1	0	0	0
	Diltiazem	0	1	0	0	0
10× C_max_	Dofetilide	2	0.0373 (0)	0.0580 (0)	0.9047 (1)	0.1326 (0)
	Bepridil	2	0	0	1	0
	Sotalol	2	0	0.712 (0)	0.288 (1)	0.712 (0)
	Quinidine	2	0	0	1	0
	Cisapride	1	0	0.728 (1)	0.272 (0)	0.272 (0)
	Terfenadine	1	0	1	0	0
	Ondansetron	1	0	1	0	0
	Chlorpromazine	1	0.9945 (1)	0.0055 (0)	0	0.9945 (1)
	Verapamil	0	0.3075 (1)	0.6925 (0)	0	0.6925 (0)
	Ranolazine	0	1	0	0	0
	Mexiletine	0	1	0	0	0
	Diltiazem	0	1	0	0	0

*The TdP risk levels were assigned category values of 2 (High), 1 (Intermediate), and 0 (Low). A classifier was trained on 11 of 12 drugs and then used to predict the category probabilities [P(x), where x is the category value] and to obtain an overall prediction error for the remaining drug (see section Cross Validation). Uncertainty model simulations were used for training and prediction. For comparison, probabilities, and prediction errors from Dutta et al. (2017) are shown in parentheses when they differed from uncertainty results*.

**Figure 7:**
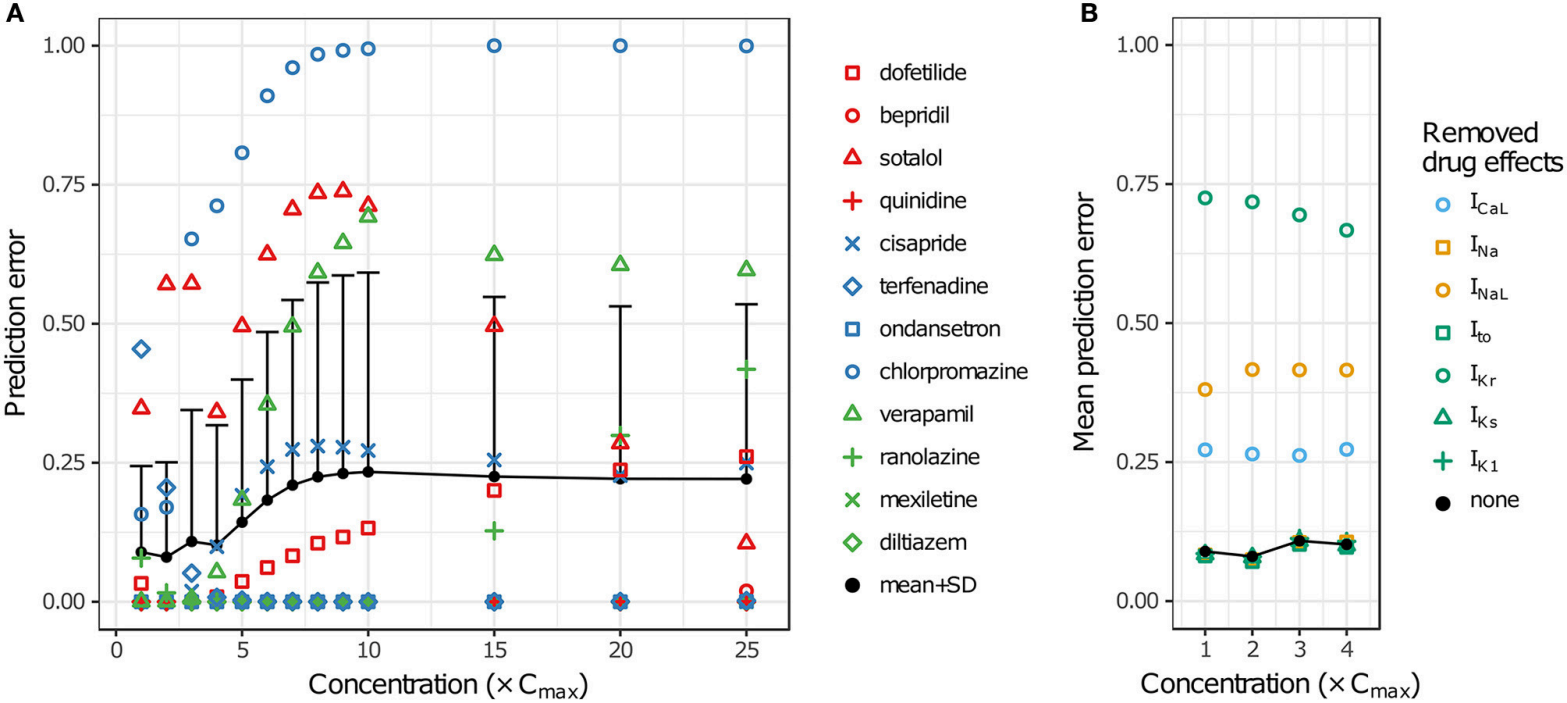
Cross validation of TdP risk stratification with uncertainty quantification. LOOCV was performed at each concentration to assess TdP risk stratification performance. Prediction error for each drug was obtained by training on qNet distribution samples from all other drugs and calculating the mean classification error of the test drug's samples. **(A)** LOOCV at 1−25× C_max_. Markers show the prediction errors for each drug when it was “left out,” as indicated in the legend. Black points and error bars are the mean + standard deviation (SD) of prediction errors at each concentration. High TdP-risk drugs are in red, Intermediate-risk drugs are in blue, and Low-risk drugs are in green. **(B)** LOOCV at 1−4× C_max_ was repeated with the drug effects for a particular ionic current removed. Black points are the mean prediction errors from **(A)**. Markers show the mean prediction errors that resulted when drug effects on the ionic current indicated in the legend were omitted from simulations.

LOOCV results for the 12 training drugs at 1–25× C_max_ are summarized in Figure 7A. As concentration increased, prediction errors improved for some drugs and worsened for others. Terfenadine's prediction error was the highest of all drugs at 1× C_max_ (0.4545) but decreased to <0.01 at 4× C_max_ (blue diamonds). On the other hand, prediction errors for chlorpromazine (blue circles), sotalol (red triangles), verapamil (green triangles), cisapride (blue× s), and dofetilide (red squares) all generally increased from 1 to 10× C_max_. Above 10× C_max_, prediction errors for dofetilide and ranolazine (green crosses) increased, while prediction errors for sotalol decreased. As a result of these trends, both the mean and the standard deviation of prediction errors were lowest at 1–4× C_max_ (Figure 7A, black points and error bars), near the concentrations for which experimental data on all currents were available for the 12 training drugs.

To explore the impact of different ionic currents on TdP risk stratification, LOOCV was repeated for a set of simulations in which drug effects on a particular ion channel were removed. This analysis was limited to 1–4× C_max_ in order to avoid concentrations at which uncertainty was due primarily to the lack of experimental data. When drug effects on I_Na_, I_to_, I_Ks_, or I_K1_ were removed, prediction errors were virtually unchanged (Figure 7B). However, when drug effects on I_CaL_, I_NaL_, or I_Kr_ were removed, prediction errors increased dramatically, indicating that TdP risk stratification of the 12 CiPA training compounds depended primarily on the drug effects for these three currents. Because most of the training compounds (other than quinidine) did not block I_Na_, I_to_, I_Ks_, or I_K1_ substantially at 1−4× C_max_, their resulting impact on TdP risk stratification was expected to be minimal.

## Discussion

Although many potential sources of uncertainty exist within the CiPA paradigm, the primary concern for the *in silico* component is uncertainty related to *in vitro* measurements of pharmacological effects on ionic currents. This study presents methods for conducting UQ within the framework of the CiPA *in silico* assay. Previously, Dutta et al. (2017) showed that the metric qNet, derived from fixed-input AP simulations incorporating multiple ion channel pharmacology, could be used to stratify the CiPA training set of 12 compounds by relative TdP risk. This study examined the impact of uncertainty in drug effects on simulation predictions. Bootstrapping and Bayesian inference were used to estimate the joint probability distributions of drug parameters in order to quantify the variability in mean drug effects. This variability was then propagated to a set of uncertainty-input AP simulations to assess the robustness of risk stratification with qNet. UQ revealed that some drug effects were insufficiently constrained at higher concentrations to be able to stratify TdP risk with high confidence. Near therapeutic concentrations, however, TdP risk stratification was robust to the uncertainty in drug effects. This study illustrates the benefits of applying UQ under the CiPA paradigm, both during model validation and when model-based predictions are used in regulatory decision making.

UQ helped to identify challenges concerning model calibration and parameter identification that will inform future model development. Such issues are frequently encountered in models of cardiac electrophysiology but are not often addressed during model development (Fink and Noble, 2009; Shotwell and Gray, 2016). In the Li et al. (2017) I_Kr_ Markov model, drug-hERG binding kinetics was characterized by six parameters, but one parameter (drug trapping rate, K_t_) was fixed at a value of 3.5× 10^−5^ ms^−1^. UQ revealed that three of the remaining five parameters (K_max_, EC_50_^n^, and V_halftrap_) could not be precisely estimated based on the available data. Although the current model structure was designed to allow for both linear and sigmoidal drug binding as well as drug trapping, this flexibility comes at the expense of parameter identifiability and presents difficulties for UQ. To address these issues, model recalibration and/or simplification may be warranted, as was done for a model of I_Na_ inactivation in Pathmanathan et al. (2015).

On the other hand, for some drugs, the observed hERG block kinetics could not be accurately captured by the I_Kr_ Markov model. For instance, at 10 nM cisapride, hERG block developed more slowly in the experimental traces than in fitted model, even when uncertainty was considered (Figure S4B). This suggests that alternative (and possibly more complex) model structures might be needed to characterize certain drugs. Thus, the challenge for CiPA is to define a one-size-fits-all model that is simple enough to be estimable but still accurate enough to predict TdP risk. The current approach attempts to strike an appropriate balance between the two concerns, combining the flexible dynamic representation of I_Kr_ block with a simplified pore-block approach for other currents. The final assessment of the model will depend on its validation with an additional 16 compounds, which will determine its suitability for CiPA (Colatsky et al., 2016).

Many IC_50_-values could not be reliably estimated from the current data, an issue raised previously by Johnstone et al. (2016a). This occurred when fitted IC_50_-values were well above the tested concentrations, resulting in high levels of uncertainty in the upper concentration ranges simulated by Li et al. (2017) and Dutta et al. (2017). The impact of this uncertainty is illustrated in results for the High-risk drug dofetilide, which is known to be a selective hERG blocker. Because its hERG selectivity could not be confirmed above 3× C_max_ with the current dataset (see Figure 3D and Figures S12–S15), uncertainty-input simulations of dofetilide above 10× C_max_ resulted in highly variable qNet-values, including very “safe” values similar to Low-risk drugs (Figure 6B). Although the impact of dofetilide on non-hERG currents is likely small, such assumptions cannot be made for new compounds, particularly if such currents and higher concentration ranges are deemed relevant for TdP risk prediction. To avoid these assumptions, *in silico* model predictions should be limited to concentrations less than or equal to the highest tested experimentally, unless the amount of drug block can be reliably extrapolated from data at lower concentrations (generally, if >60% block is achieved experimentally, see Figure 4). Thus, UQ highlights the importance of obtaining the appropriate data for generating reliable model predictions within the CiPA paradigm. For the current training set, TdP risk prediction appeared to depend solely on I_CaL_, I_NaL_, and I_Kr_ data (Figure 7B), so this “60% rule” may potentially only need apply to these three currents. However, the importance of I_Na_, I_to_, I_Ks_, and I_K1_ cannot be discounted entirely because most of the training compounds did not substantially affect these currents. Further sensitivity analysis of qNet and testing with additional compounds may provide insight into the importance of these currents for TdP risk prediction.

Hierarchical UQ approaches may account for some of the discrepancies between observed experimental variability and the estimated variability of model outputs in the present study. For example, at the highest bepridil concentration (300 nM), the kinetics of I_Kr_ block in a few cells was noticeably faster than that of other cells and the fitted bootstrap traces. Although it is unlikely that any single method could capture all observed variability, hierarchical approaches to quantify inter-individual variability may provide a more accurate representation of the true physiological variability than do population-averaged approaches (Pathmanathan et al., 2015). Recently, Johnstone et al. (2016a) used a hierarchical statistical model to assess the inter-experiment variability of drug block data from Crumb et al. (2016). Such an approach could be explored in the future if complete dose-response data for all ionic currents become available. In the present study, however, the IC_50_ of most currents could not be reliably estimated, so a further hierarchical analysis was not warranted. For the Li et al. (2017) I_Kr_ Markov model, hierarchical methods would be more experimentally and computationally challenging. Experimentally, this would require obtaining hERG block data for each cell at multiple concentrations in order to estimate individual dose-dependent kinetics. However, due to stability and time limitations associated with the current experimental protocol, cells were only recorded at a single concentration. The computational demands of estimating hierarchical model parameters for dynamic models would also be very high because of the need to integrate differential equations. Addressing these difficulties may be unnecessary for CiPA, however, if a population-averaged approach to UQ is shown to provide sufficient information for robust TdP risk prediction.

The UQ results presented in this study illustrate the need to evaluate model predictions in the context of uncertainty. Previously, Dutta et al. (2017) demonstrated that qNet could separate the CiPA training drugs by TdP risk better than metrics based on AP or Ca^2+^ transient morphology. In addition, the mean LOOCV prediction error of qNet was lower when drugs were simulated at 10× and 20× C_max_ than at 1× C_max_, suggesting that higher concentrations could provide better risk separation. However, this assessment was based only on fixed-input simulations. When uncertainty inputs were used to classify drugs, mean LOOCV prediction error was lowest at 1–4× C_max_ and worsened as concentration increased above 4× C_max_ (Figure 7A). In part, the differences in LOOCV results for fixed vs. uncertainty inputs were due to the high uncertainty in qNet for drugs such as, dofetilide and verapamil above 4× C_max_ (Figure 6C). However, these differences also arose because when uncertainty was low, classification with qNet probability distributions was more robust than with fixed qNet-values, which improved the mean LOOCV prediction error at 1× C_max_ (Table 5). UQ also provided an indication of the degree to which drugs could be separated, so LOOCV was more sensitive to subtle changes in qNet. Risk stratification of the training drugs at >4× C_max_ may be improved if additional *in vitro* data are obtained at higher concentrations and incorporated into the model. However, it is important to keep in mind that the CiPA-assigned TdP risk levels for the 12 training and 16 validation compounds are not absolute; these relative risks are mainly based on years of clinical evidence and expert opinion rather than a quantitative measure of real-world data. Effort is ongoing within the CiPA framework to develop more objective and quantitative TdP risk categorization systems based on postmarket data, which will help to refine the model and metric for more accurate TdP risk assessment.

This study did not address the issue of model uncertainty related to physiological variability because the focus of CiPA is on drug screening and obtaining an estimate of proarrhythmic risk that can be used to assess overall drug safety, not on predicting risk in specific individuals or subpopulations. However, this is an important topic for many safety pharmacology applications involving mathematical modeling. In pharmacokinetics, non-linear mixed effects (NLME) models have routinely been applied to quantify intersubject variability (Fitzmaurice et al., 2008). However, methods for quantifying physiological variability in more complex cardiac electrophysiology models are not well-established. One approach has been to use a “population” of *in silico* cardiac cell models, generated by randomly varying model parameters, to explore mechanisms underlying physiological variability and to predict the resulting variability in drug responses, such as, hERG block-induced changes in APD (Sarkar and Sobie, 2011; Britton et al., 2013). The aim of UQ is to estimate model parameters within a statistical framework and then to give probabilistic predictions. Pathmanathan et al. (2015) used data from 10 to 16 cells and NLME modeling to perform a thorough UQ analysis of a single model parameter, steady-state I_Na_ inactivation. But applying similar approaches to whole cell models, which typically have dozens of parameters, would require large amounts of data and, most likely, simpler models, as discussed by Pathmanathan et al. (2015). Nevertheless, such studies on physiological variability can be considered in complement with the results in this study concerning UQ of drug effects, providing insight into how multiple sources of uncertainty may impact variability in drug responses.

One additional issue that was not explored in this study was the effect of the number of experimental repeats on parameter uncertainty. For the manual patch clamp data used in this study, 4–10 repeats were obtained per drug concentration for the hERG experiments, and 3–4 repeats were obtained for non-hERG experiments. Thus, based on the current dataset, 3–4 experimental repeats appeared sufficient to constrain the model parameters for TdP risk prediction. However, data obtained from multiple labs or using automated, high-throughput systems can be much more variable, and more experimental repeats may be needed to accurately estimate the mean drug effect with these types of data (Elkins et al., 2013). These issues may be addressed in the future CiPA *in silico* validation phase.

In summary, risk stratification of the CiPA training drugs with the currently available data was most reliable near the maximum clinical concentration. This was because most of the *in vitro* experiments were designed around known therapeutic concentrations that often did not block the major ionic currents, and measurements at significantly higher concentrations were not consistently obtained for all drugs. The lack of experimental data produced a large degree of uncertainty in drug effects, which negatively impacted the ability to distinguish between drugs of different TdP risk at higher concentrations. Hence, our findings suggest that for new compounds, the CiPA *in silico* assay will require *in vitro* measurements at much higher drug concentrations that can achieve significant ionic current block if the model is expected to provide TdP risk predictions with high confidence. Whether this will be necessary for all seven ion channels that have been suggested as part of CiPA, however, remains to be determined.

## Author contributions

KC designed and carried out the study and wrote the manuscript. SD, GM, KB, and ZL contributed to the study design and analysis. ZL supervised the project. ZL, DS, SD, GM, KB, and TC revised the manuscript. JS, PT, MW, and WW collected the data and provided guidance on interpretation of the data.

### Conflict of interest statement

The authors declare that the research was conducted in the absence of any commercial or financial relationships that could be construed as a potential conflict of interest.
