# Supplementary material for: Uncertainty Quantification Reveals the Importance of Data Variability and Experimental Design Considerations for in Silico Proarrhythmia Risk Assessment
Source: Front Physiol. 2017 Nov 21;8:917. doi: 10.3389/fphys.2017.00917 (PMC5702340; doi:10.3389/fphys.2017.00917)
Supplement: Supplementary file 1 [file DataSheet1.PDF]

# *Supplementary Material*: Uncertainty quantification for *in silico* proarrhythmia risk assessment under the CiPA initiative

**Kelly C. Chang, Sara Dutta, Gary R. Mirams, Kylie A. Beattie, Jiansong Sheng, Phu N. Tran, Min Wu, Wendy W. Wu, Thomas Colatsky, David G. Strauss, and Zhihua Li***

*Correspondence:
Author Name: Zhihua Li
Zhihua.Li@fda.hhs.gov

## 1 SUPPLEMENTARY METHODS

### 1.1 Bootstrap fitting

The `cmaes` package (Hansen, 2006; Trautmann et al., 2011) was used to refit the drug-hERG kinetics parameters of the Li et al. (2017) $I_{Kr}$ Markov model for each bootstrap sample (see main text). To aid in the optimization process, parameters were encoded to identical ranges for the CMA-ES algorithm to explore, as recommended on the website `https://www.lri.fr/~hansen/cmaes_inmatlab.html`. All parameters were encoded logarithmically from their selected ranges [a, b] (listed in Table S5) to the range [0, 10], with the equation:

$$f(x) = a\left(\frac{b}{a}\right)^{x/10}$$

The number of offspring (`lambda`) was set to 80 and the stopping tolerance (`stop.tolx`) was set to $10^{-3}$; defaults were used for all other settings.

### 1.2 Markov-chain Monte Carlo simulation

Markov-chain Monte Carlo (MCMC) simulation was performed with the `FME` package (Soetaert and Petzoldt, 2010) to estimate uncertainty in Hill equation parameters. MCMC simulation was performed using the following settings: `f` was the function of model residuals, initial values were set to the best-fit parameters (`p = mf$par`) from the optimal nonlinear least squares fit of the Hill equation (`mf`) obtained with the `modFit` function using the Levenberg-Marquardt algorithm (`method = "Marq"`). The initial proposal covariance was set to the scaled parameter covariance matrix of the fitted model, as recommended in the FME documentation (`jump = summary(mf)$cov.scaled*2.4^2/2`). The prior mean of the error variance $\sigma^2$ (a nuisance parameter) was set to the variance of the fitted residuals (`var0 = summary(mf)$modVariance`), and the prior accuracy parameter was set to give equal weight to the prior and current error variance (`wvar0 = 1`). Adaptation only occurred during the first 10,000 iterations, which were discarded as burn-in (`burninlength = 10000`). Following burn-in, every 10th

iteration was saved over the next 20,000 iterations (`niter = 30000, outputlength = 2000`). Convergence was evaluated with the Geweke diagnostic test (Geweke, 1992) using version 0.18-1 of the `coda` package (Plummer et al., 2006). If the Geweke test (`geweke.diag`) comparing the first 10% and the last 50% of the 2,000 saved iterations indicated a lack of convergence (Z-score for either $pIC_{50}$ or h between -1.96 and 1.96), then the burn-in/adaptation period was increased by an additional 10,000 iterations and the entire MCMC simulation was rerun. This was repeated until the absolute value of Z-scores was $>$1.96.

# 2 SUPPLEMENTARY TABLES AND FIGURES

## 2.1 Tables

**Table S1.** Parameter values for the physiological component of the Li et al. (2017) $I_{Kr}$ Markov model.

| State transition | A ($ms^{-1}$) | B ($mV^{-1}$) | q |
|---|---|---|---|
| 1 | 0.0264 | 4.63e-05 | 4.843 |
| 2 | 4.99e-06 | -0.00423 | 4.23 |
| 3 | 0.001214 | 0.008516 | 4.962 |
| 4 | 1.85e-05 | -0.04641 | 3.769 |
| 11 | 0.000787 | 1.54e-08 | 4.942 |
| 21 | 5.46e-06 | -0.1688 | 4.156 |
| 31 | 0.005509 | 7.77e-09 | 4.22 |
| 41 | 0.001416 | -0.02877 | 1.459 |
| 51 | 0.4492 | 0.008595 | 5 |
| 52 | 0.3181 | 3.61e-08 | 4.663 |
| 53 | 0.149 | 0.004668 | 2.412 |
| 61 | 0.01241 | 0.1725 | 5.568 |
| 62 | 0.3226 | -0.00066 | 5 |
| 63 | 0.008978 | -0.02215 | 5.682 |

**Table S2.** Conductance scaling factors for the optimized $I_{Kr}$-dynamic ORd model (Dutta et al., 2017). Maximal current conductances were multiplied by the corresponding scaling factor.

| Conductance | Scaling factor |
|---|---|
| GKr | 1.0127 |
| GKs | 1.870 |
| GK1 | 1.698 |
| PCa | 1.007 |
| GNaL | 2.661 |

**Table S3.** Steady-state values for the optimized $I_{Kr}$-dynamic ORd model (Dutta et al., 2017) paced at a cycle length of 2 s.

| Parameter | Value |
|---|---|
| v | -88.0145 mV |
| nai | 6.46961 mM |
| nass | 6.46967 mM |
| ki | 145.501 mM |
| kss | 145.501 mM |
| cai | 7.45e-05 mM |
| cass | 7.30e-05 mM |
| cansr | 1.37897 mM |
| cajsr | 1.37944 mM |
| m | 0.007335 |
| hf | 0.698542 |
| hs | 0.698542 |
| j | 0.698542 |
| hsp | 0.455526 |
| jp | 0.698541 |
| mL | 0.000188 |
| hL | 0.513396 |
| hLp | 0.307923 |
| a | 0.001 |
| iF | 0.999555 |
| iS | 0.871715 |
| ap | 0.00051 |
| iFp | 0.999555 |
| iSp | 0.905466 |
| d | 2.33e-09 |

| Parameter | Value |
|---|---|
| ff | 1 |
| fs | 0.971897 |
| fcaf | 1 |
| fcas | 1 |
| jca | 1 |
| nca | 0.001539 |
| ffp | 1 |
| fcafp | 1 |
| xrf | 8.00e-06 |
| xrs | 0.160765 |
| xs1 | 0.130941 |
| xs2 | 0.000193 |
| xk1 | 0.996756 |
| Jrelnp | 1.47e-07 mM/ms |
| Jrelp | 1.83e-07 mM/ms |
| CaMKt | 0.003252 |
| IC1 | 0.999637 |
| IC2 | 6.83e-05 |
| C1 | 1.80e-08 |
| C2 | 8.27e-05 |
| O | 0.000156 |
| IO | 5.68e-05 |
| IObound | 0 |
| Obound | 0 |
| Cbound | 0 |

**Table S4.** Maximum therapeutic concentration ($C_{max}$) for the 12 CiPA training drugs.

| Drug | $C_{max}$ (nM) | Reference |
|---|---|---|
| Dofetilide | 2 | Redfern et al. (2003) |
| Bepridil | 33 | Redfern et al. (2003) |
| Sotalol | 14690 | Kramer et al. (2013) |
| Quinidine | 3237 | Redfern et al. (2003) |
| Cisapride | 2.6 | Crumb et al. (2016) |
| Terfenadine | 4 | Redfern et al. (2003) |
| Ondansetron | 139 | Li et al. (2017) |
| Chlorpromazine | 38 | Redfern et al. (2003) |
| Verapamil | 81 | Redfern et al. (2003) |
| Diltiazem | 122 | Redfern et al. (2003) |
| Ranolazine | 1948.2 | Crumb et al. (2016) |
| Mexiletine | 4129 | Redfern et al. (2003) |

**Table S5.** Bounds for the dynamic drug-hERG binding parameters used to fit bootstrap samples.

| Parameter | Lower | Upper |
|---|---|---|
| $K_{max}$ | $10^{-8}$ | $10^{8}$ |
| $K_u$ ($ms^{-1}$) | $10^{-9}$ | 1 |
| n | 0.5 | 2 |
| $EC_{50}{}^{n}$ ($nM^{n}$) | 0.001 | $10^{9}$ |
| $V_{halftrap}$ (mV) | -200 | -1 |

## 2.2 Figures

**Figures S1–11.** Uncertainty in drug-hERG kinetic parameters. **(A)** The joint probability distribution of $K_{max}$ (maximum drug effect at saturating concentrations), $K_u$ (rate of drug unbinding), n (Hill coefficient of drug binding), $EC_{50}{}^{n}$ ($n^{th}$ power of the half-maximal drug concentration), and $V_{halftrap}$ (drug trapping potential) was estimated by bootstrapping. Plots on the diagonal show the marginal histograms of each parameter (log-transformed in some cases). Plots below the diagonal show pairwise scatter plots of the fitted parameters for 2,000 bootstrap samples. **(B)** Kinetics of hERG block during 10 sweeps of a voltage-clamp protocol (Milnes et al., 2010). Shaded areas show the range of block produced by the parameters from panel **A**. Lines show the experimental results used to fit the data (down-sampled 5× for clarity).

**Figures 12–62.** Uncertainty in the dose-response relationships for current block by the 12 CiPA training drugs. Dose-response relationships were evaluated for $I_{CaL}$, $I_{K1}$, $I_{Ks}$, $I_{Na}$, $I_{NaL}$, and $I_{to}$ only if $IC_{50}$ values were defined in Li et al. (2017). **(A)** The joint distribution of $pIC_{50}$ and Hill coefficient (h) values, estimated with a Bayesian inference approach. Marginal histograms are displayed on the diagonal plots, and pairwise scatter plots are below the diagonal (2,000 samples per drug). $IC_{50}$ values are in nM. **(B)** Dose-response relationships. Solid lines show the Hill equation defined by $IC_{50}$ and h values from Li et al. (2017). Shaded areas denote the 95% CI of percentage block at each concentration, as determined by the parameters in panel **A**. Circles are the experimental values used to fit the dose-response curves. Vertical dotted lines indicate the limits of the concentration range used in AP simulations (1–25× $C_{max}$).

**(A)** Dofetilide-hERG kinetic parameters

**(B)** Current block during Milnes protocol

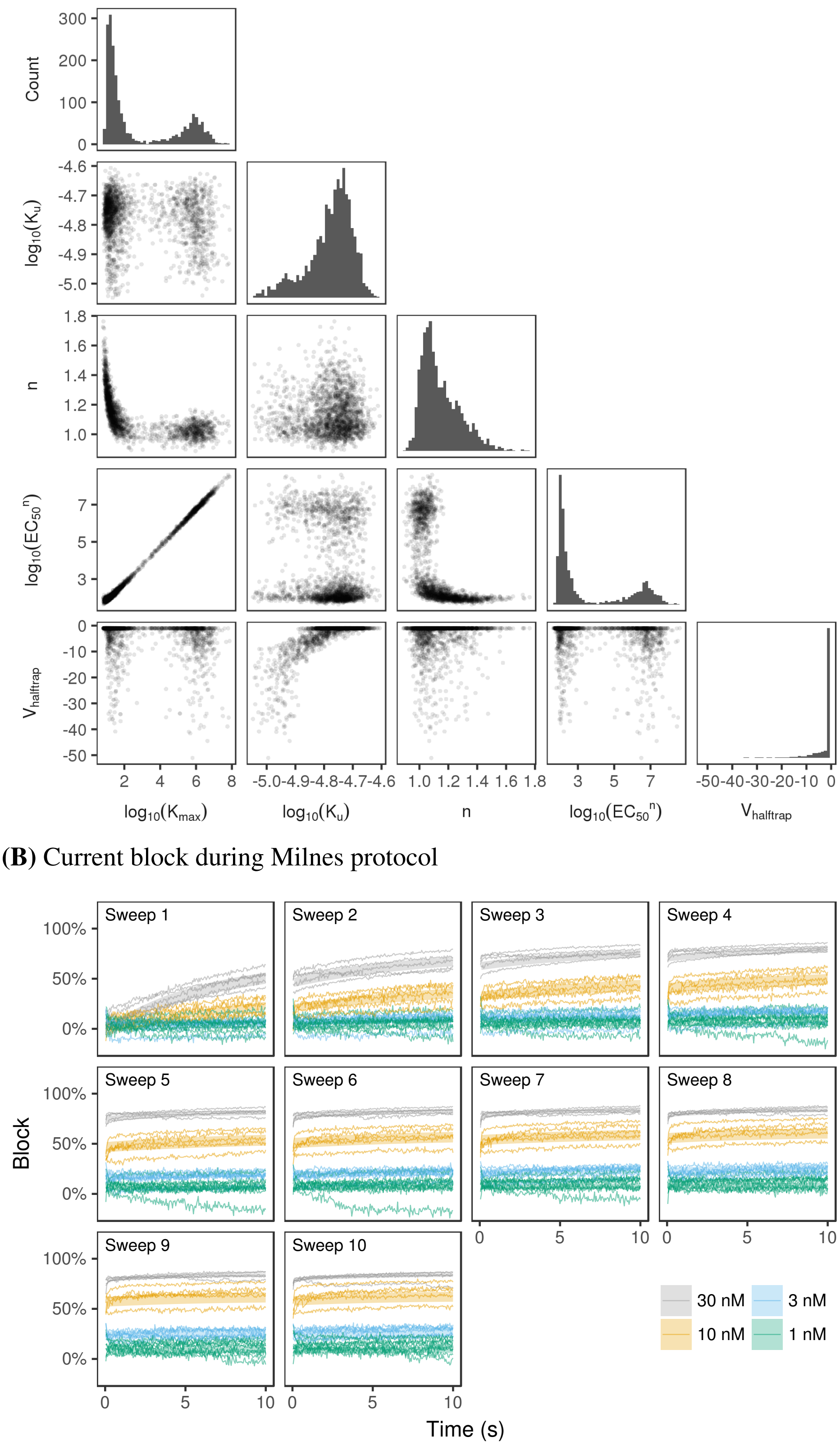


**Figure S1.** Bootstrap fitting results for dofetilide-hERG kinetic parameters.

**(A)** Sotalol-hERG kinetic parameters

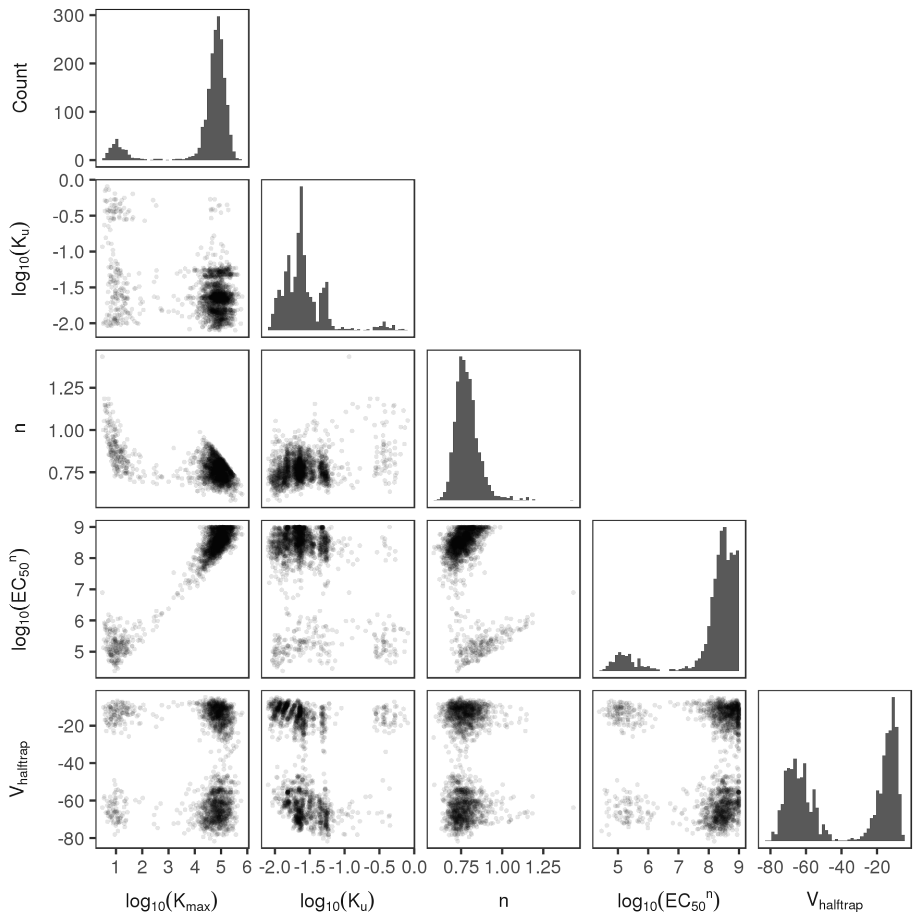


**(B)** Current block during Milnes protocol

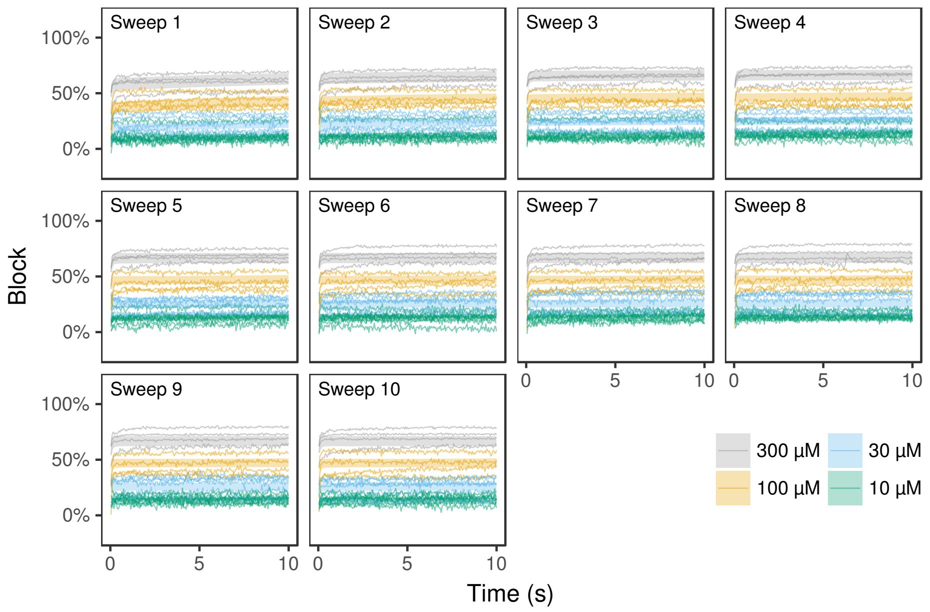


**Figure S2.** Bootstrap fitting results for sotalol-hERG kinetic parameters.

**(A)** Quinidine-hERG kinetic parameters

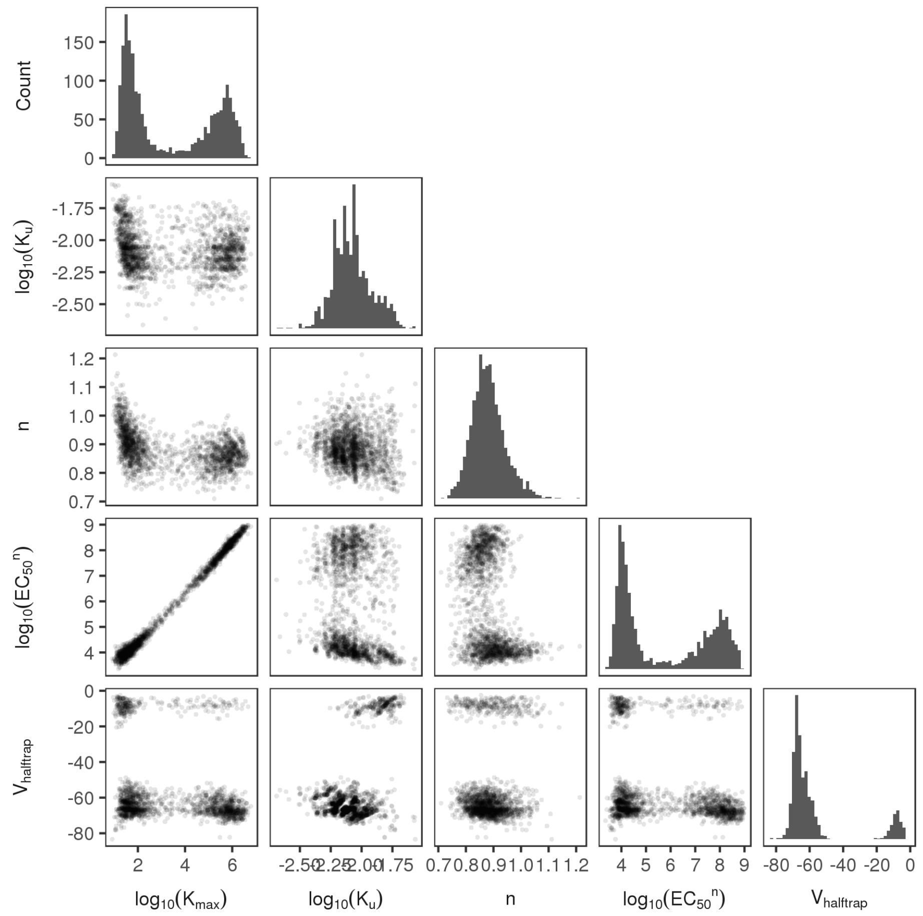


**(B)** Current block during Milnes protocol

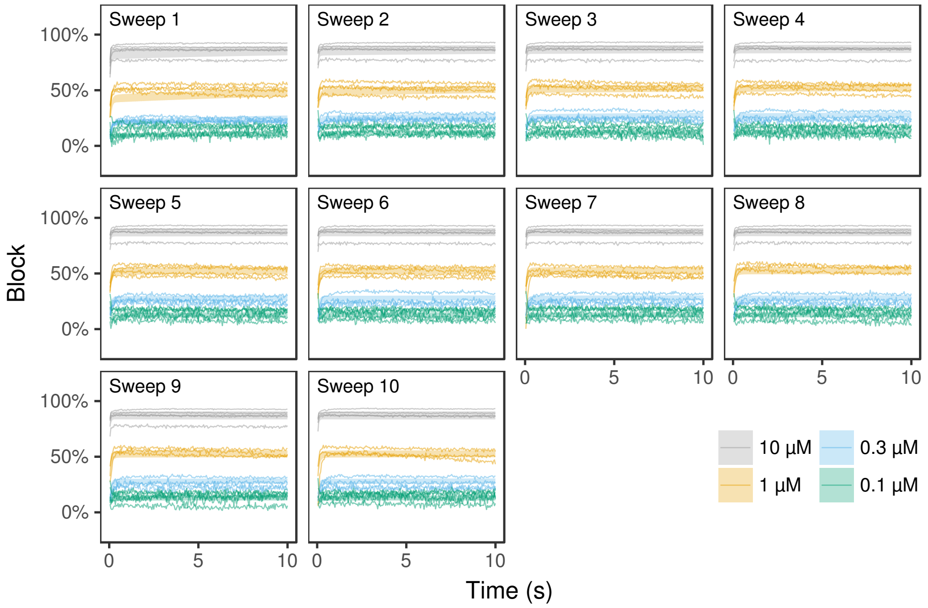


**Figure S3.** Bootstrap fitting results for quinidine-hERG kinetic parameters.

**(A)** Cisapride-hERG kinetic parameters

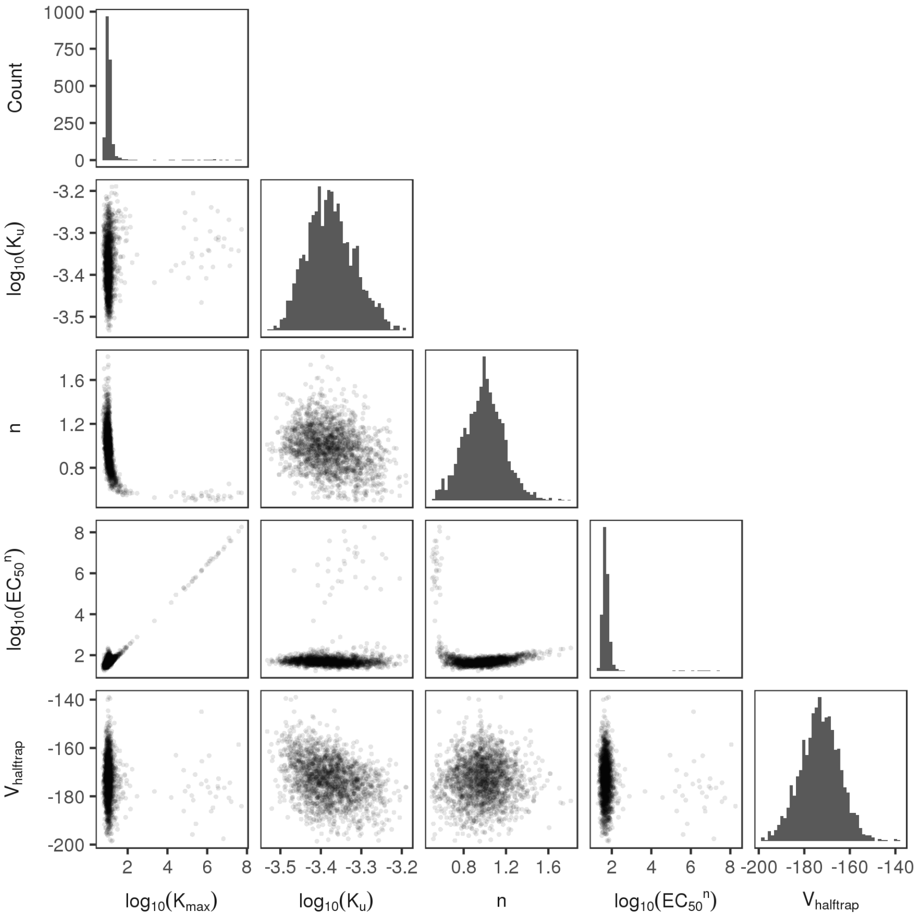


**(B)** Current block during Milnes protocol

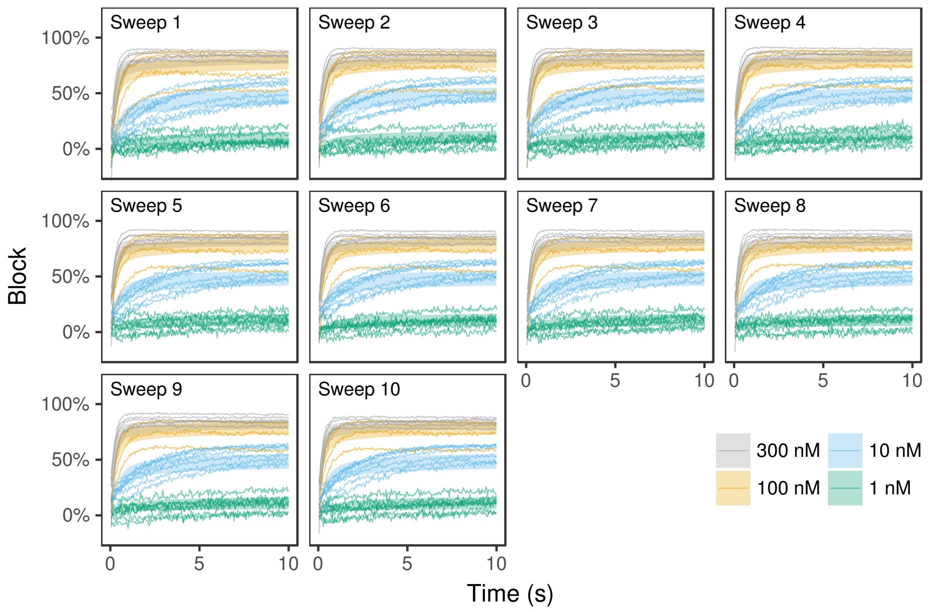


**Figure S4.** Bootstrap fitting results for cisapride-hERG kinetic parameters.

**(A)** Terfenadine-hERG kinetic parameters

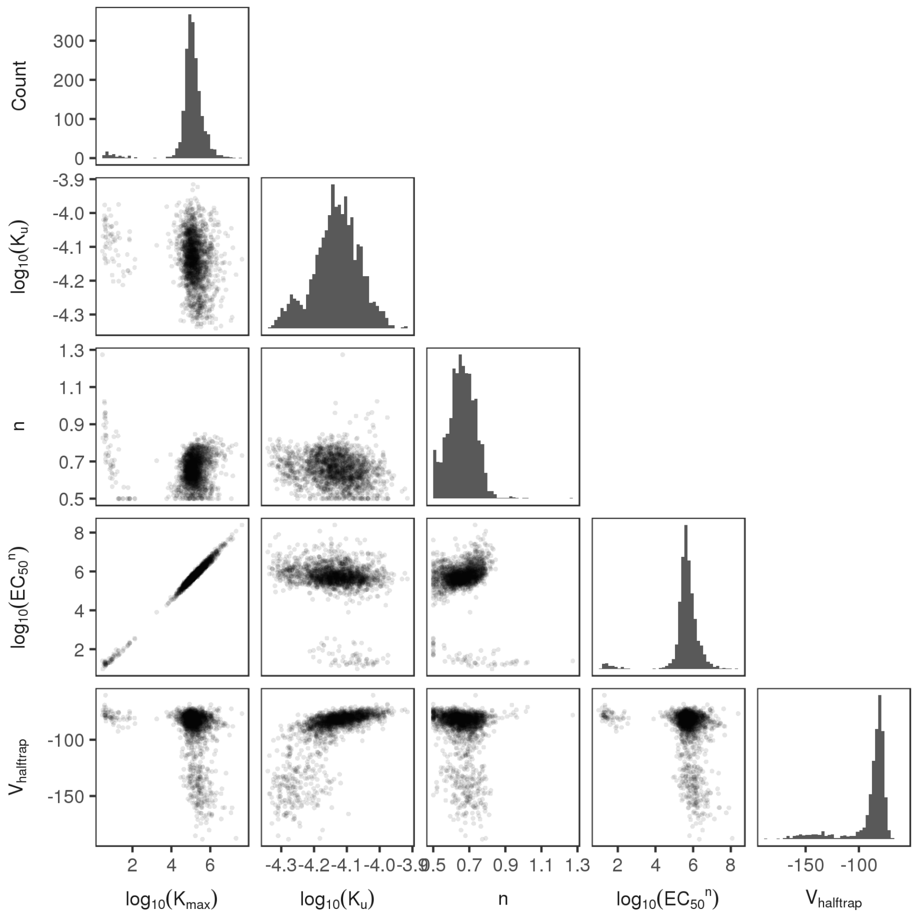


**(B)** Current block during Milnes protocol

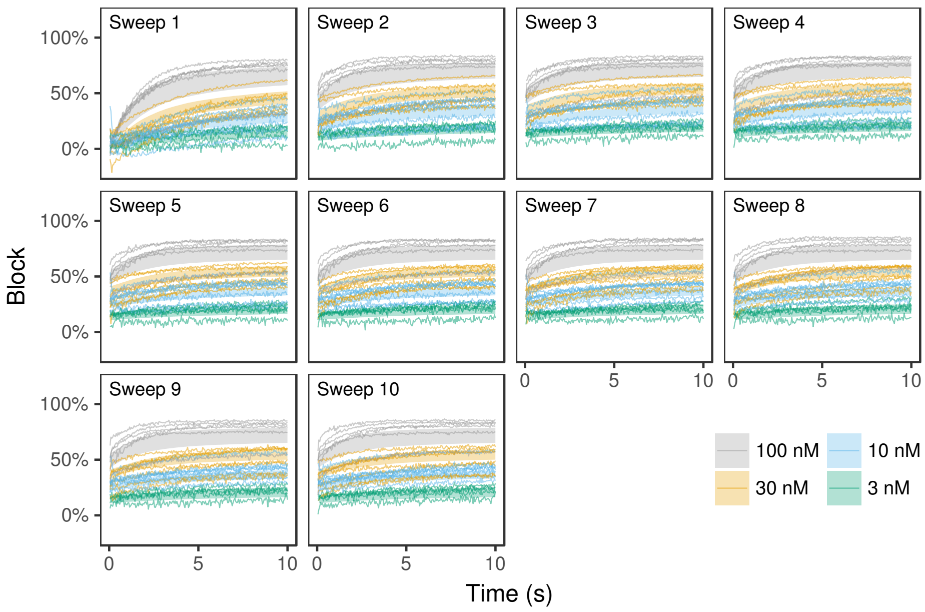


**Figure S5.** Bootstrap fitting results for terfenadine-hERG kinetic parameters.

**(A)** Ondansetron-hERG kinetic parameters

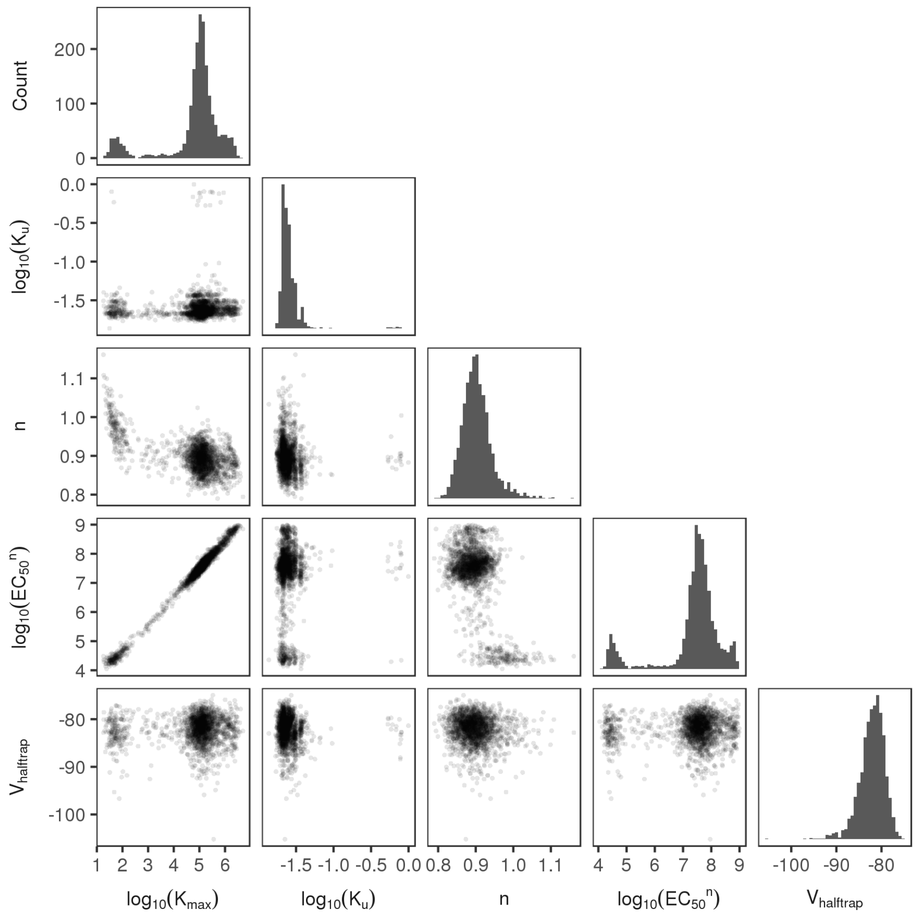


**(B)** Current block during Milnes protocol

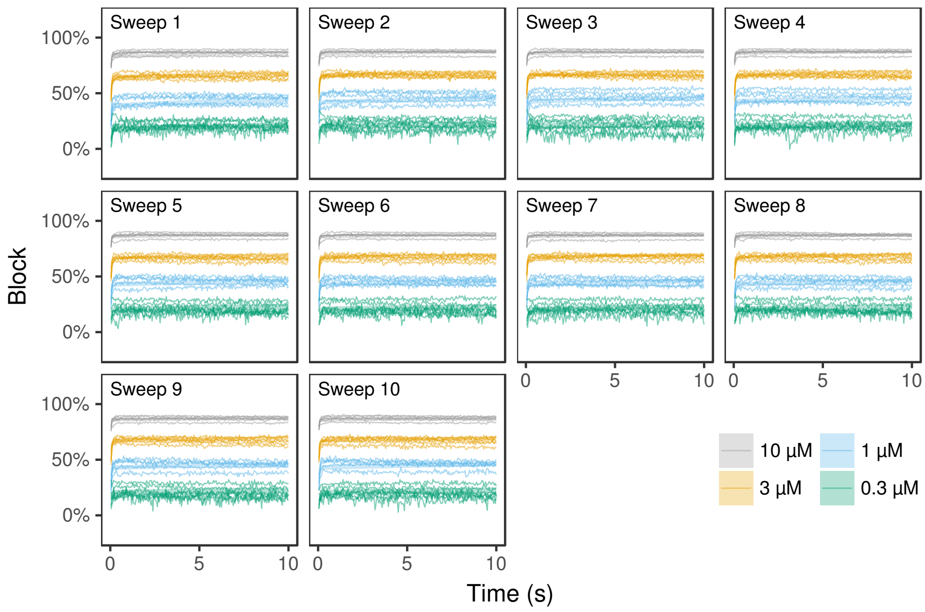


**Figure S6.** Bootstrap fitting results for ondansetron-hERG kinetic parameters.

**(A)** Chlorpromazine-hERG kinetic parameters

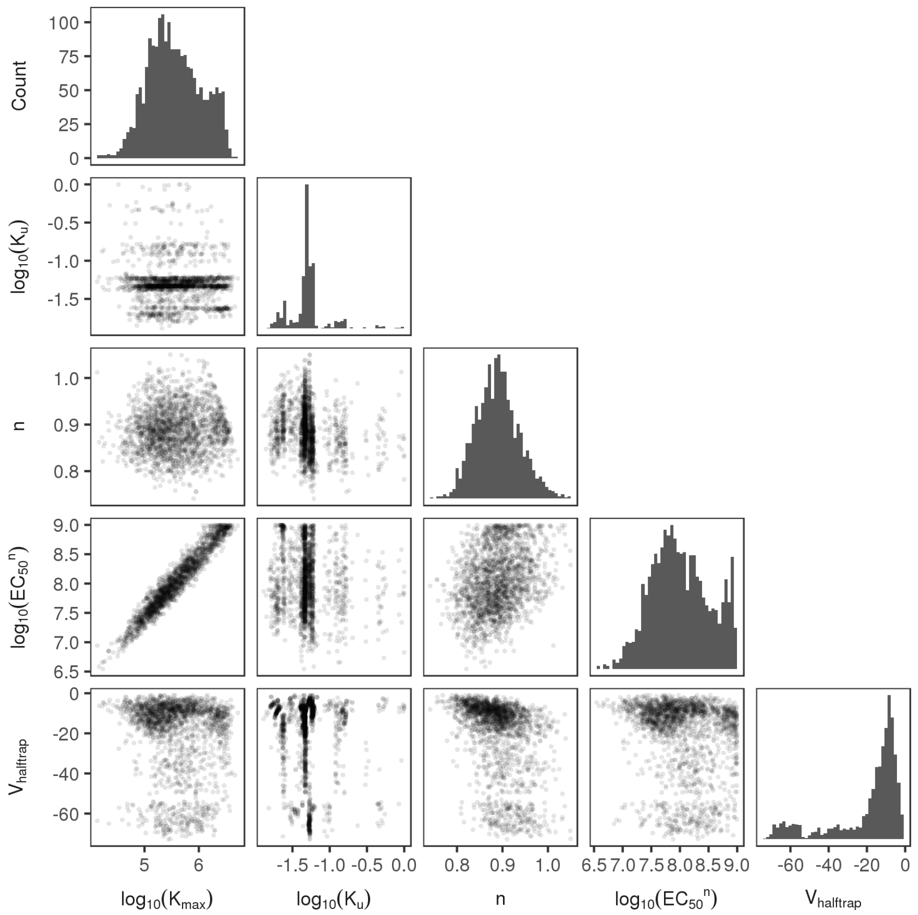


**(B)** Current block during Milnes protocol

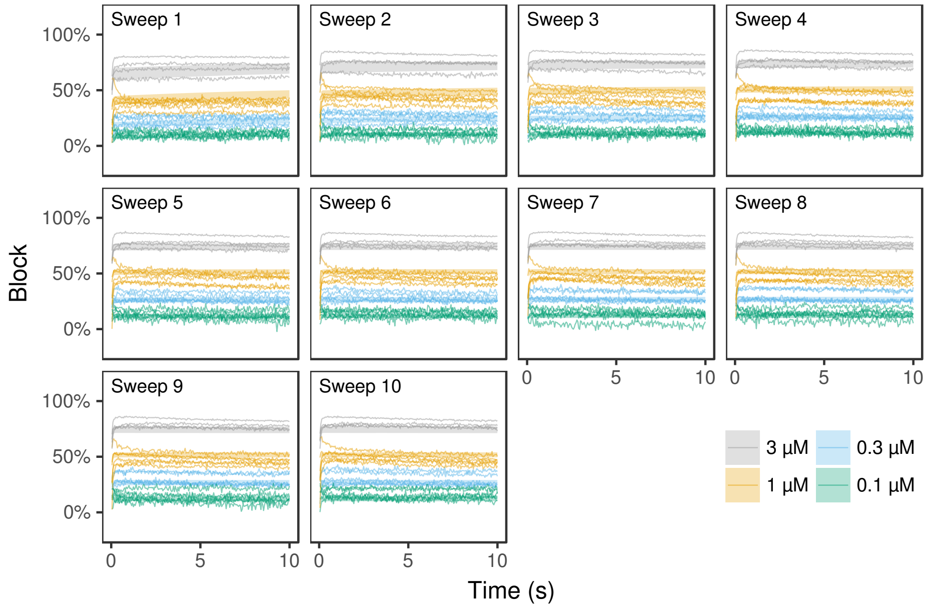


**Figure S7.** Bootstrap fitting results for chlorpromazine-hERG kinetic parameters.

**(A)** Verapamil-hERG kinetic parameters

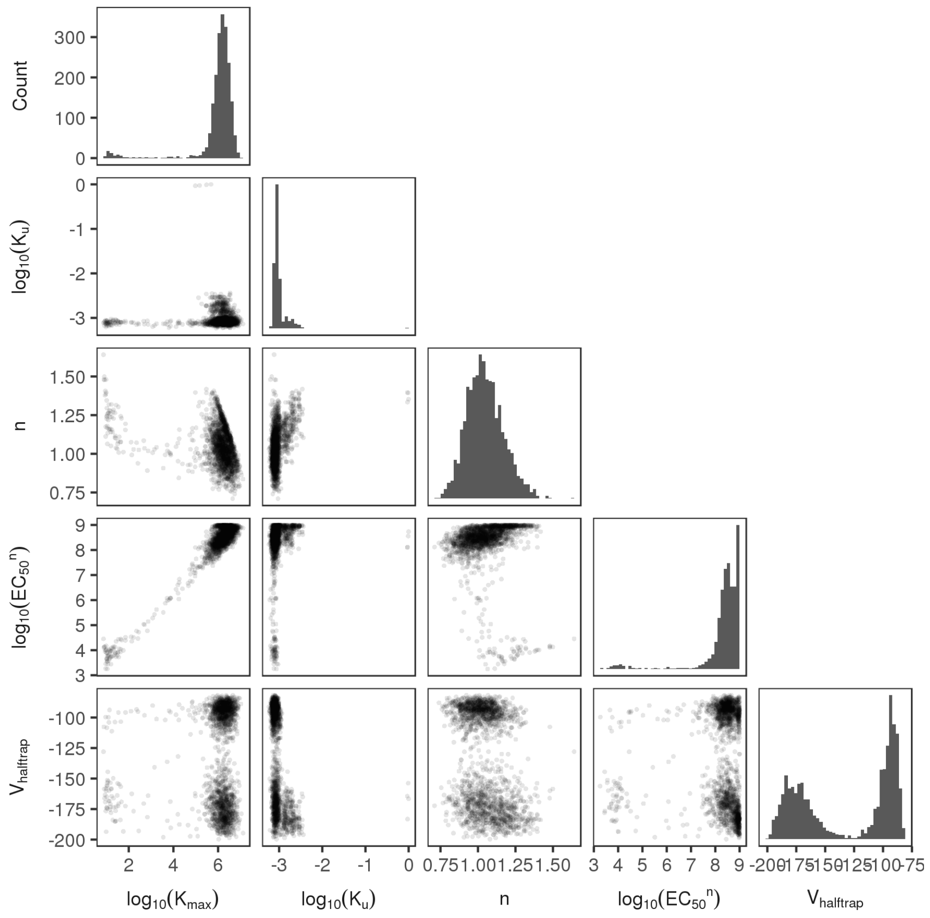


**(B)** Current block during Milnes protocol

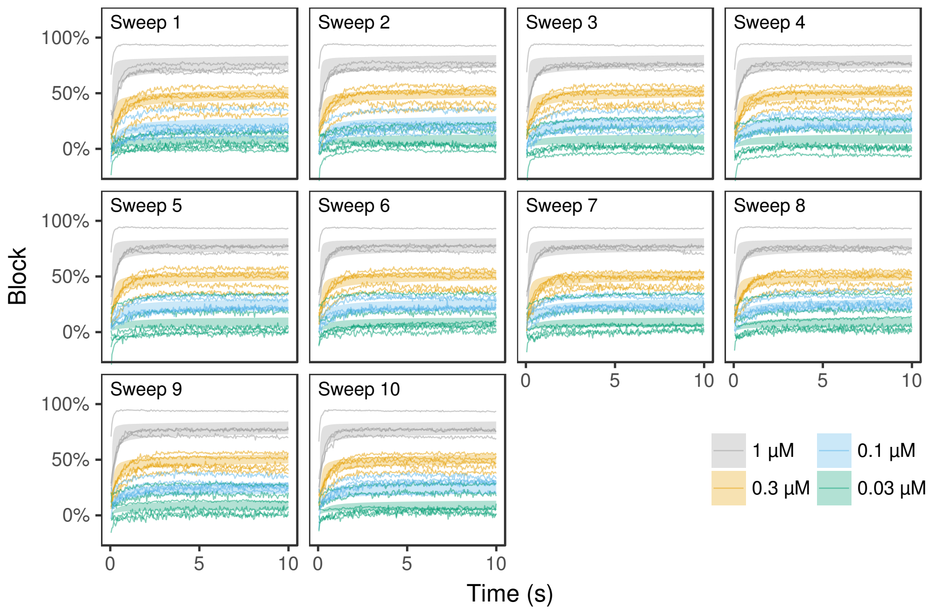


**Figure S8.** Bootstrap fitting results for verapamil-hERG kinetic parameters.

**(A)** Ranolazine-hERG kinetic parameters

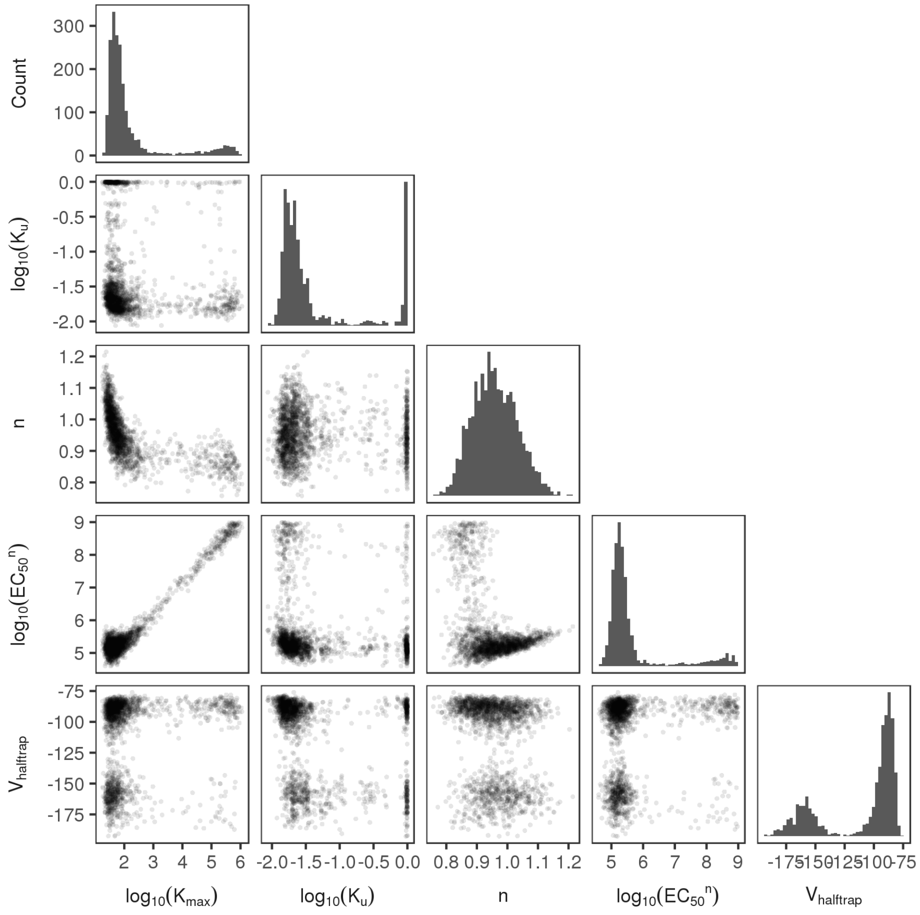


**(B)** Current block during Milnes protocol

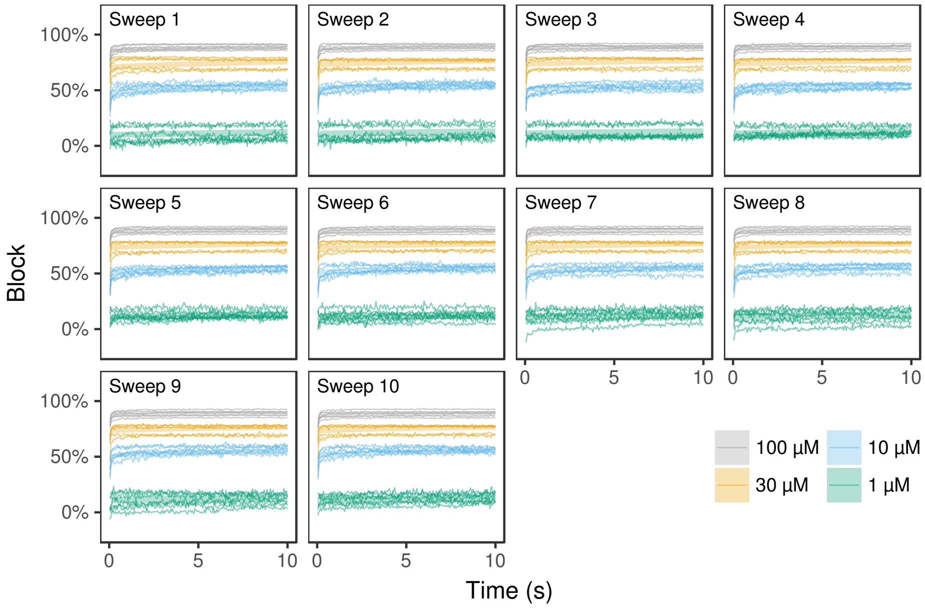


**Figure S9.** Bootstrap fitting results for ranolazine-hERG kinetic parameters.

**(A)** Mexiletine-hERG kinetic parameters

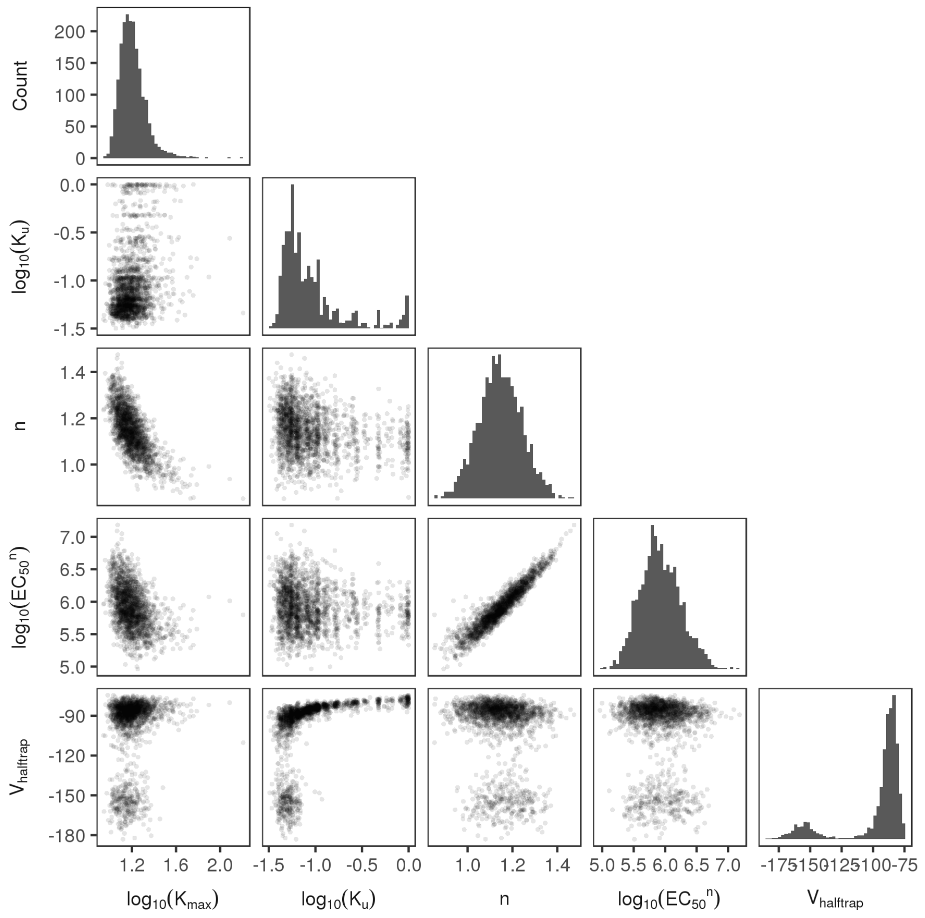


**(B)** Current block during Milnes protocol

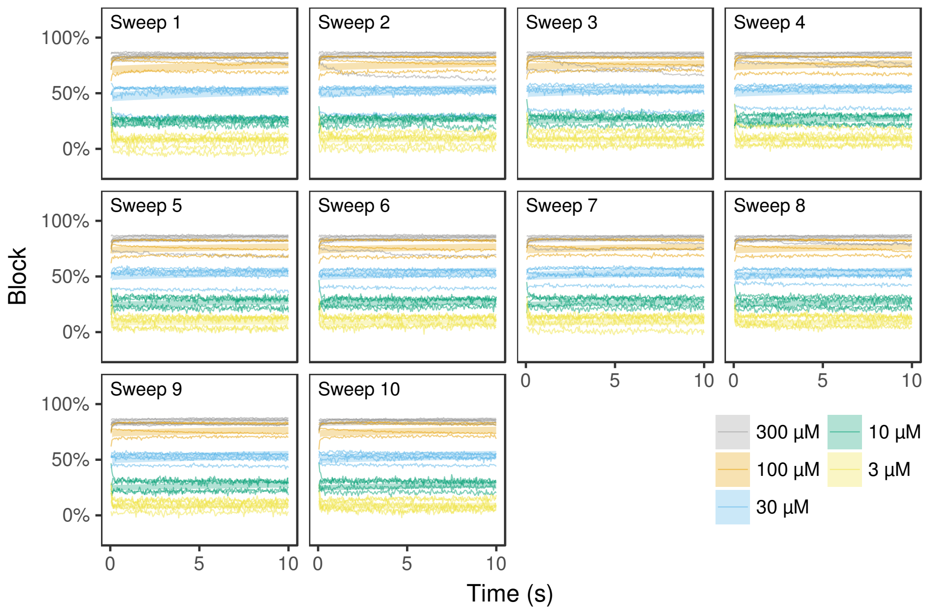


**Figure S10.** Bootstrap fitting results for mexiletine-hERG kinetic parameters.

**(A)** Diltiazem-hERG kinetic parameters

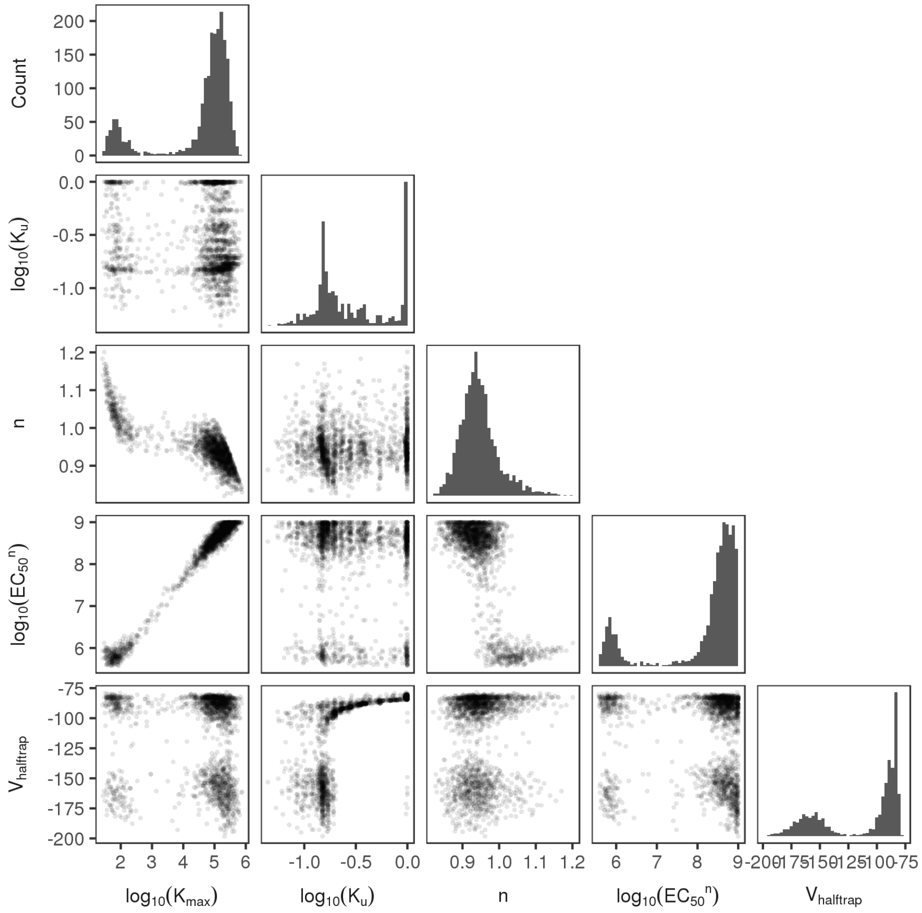


**(B)** Current block during Milnes protocol

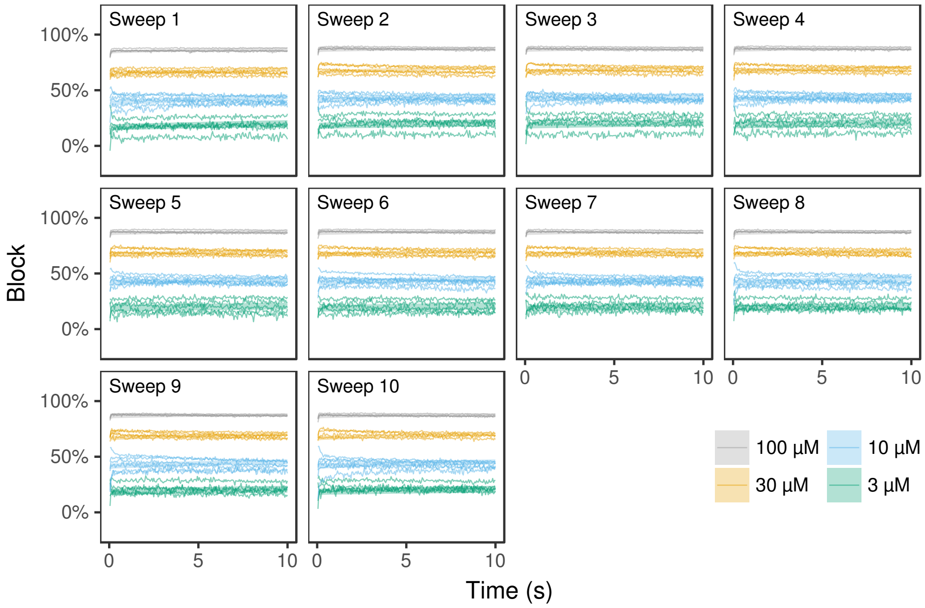


**Figure S11.** Bootstrap fitting results for diltiazem-hERG kinetic parameters.

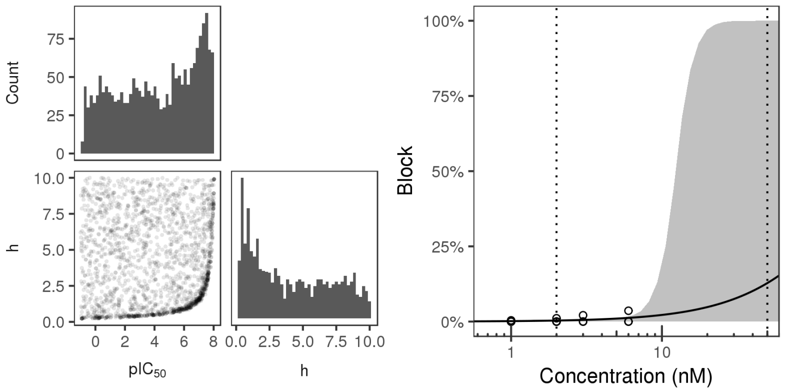


**(A)** Hill equation parameters **(B)** Dose-response curve

**Figure S12.** MCMC simulation results for dofetilide-$I_{CaL}$ Hill equation parameters.

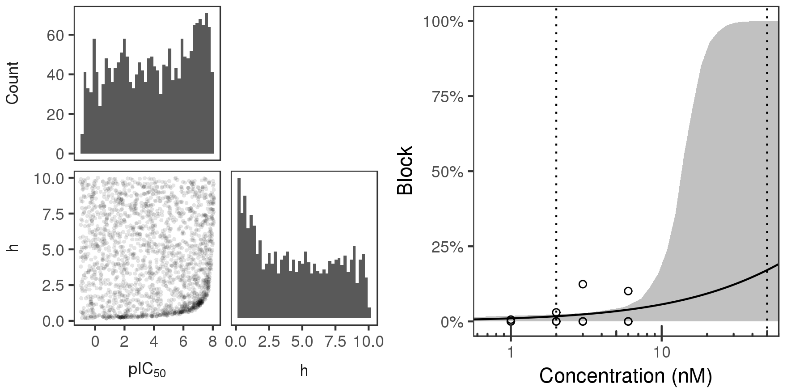


**(A)** Hill equation parameters **(B)** Dose-response curve

**Figure S13.** MCMC simulation results for dofetilide-$I_{K1}$ Hill equation parameters.

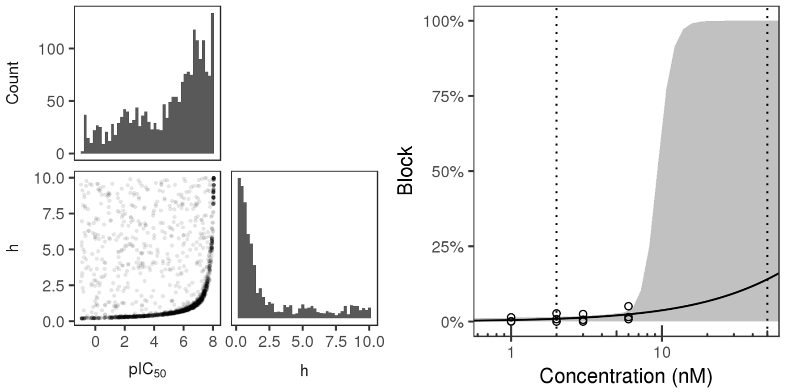


**(A)** Hill equation parameters **(B)** Dose-response curve

**Figure S14.** MCMC simulation results for dofetilide-$I_{Na}$ Hill equation parameters.

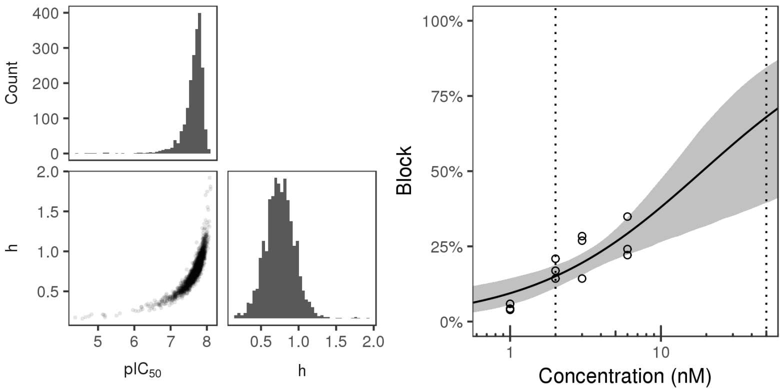


**(A)** Hill equation parameters **(B)** Dose-response curve

**Figure S15.** MCMC simulation results for dofetilide-$I_{to}$ Hill equation parameters.

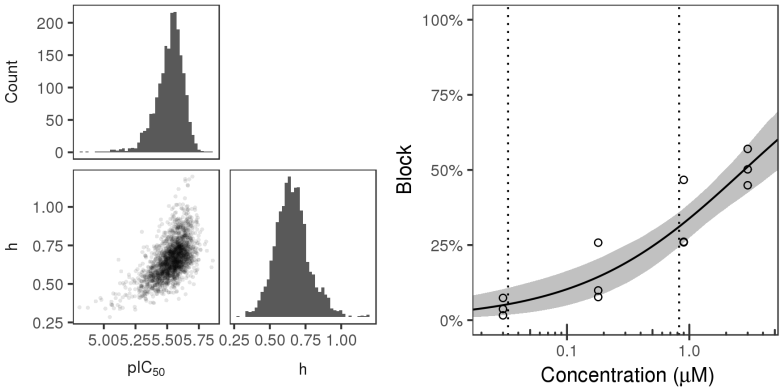


**(A)** Hill equation parameters **(B)** Dose-response curve

**Figure S16.** MCMC simulation results for bepridil-$I_{CaL}$ Hill equation parameters.

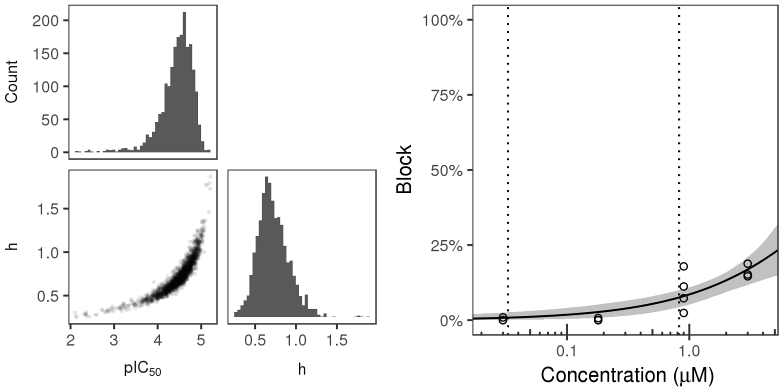


**(A)** Hill equation parameters **(B)** Dose-response curve

**Figure S17.** MCMC simulation results for bepridil-$I_{Ks}$ Hill equation parameters.

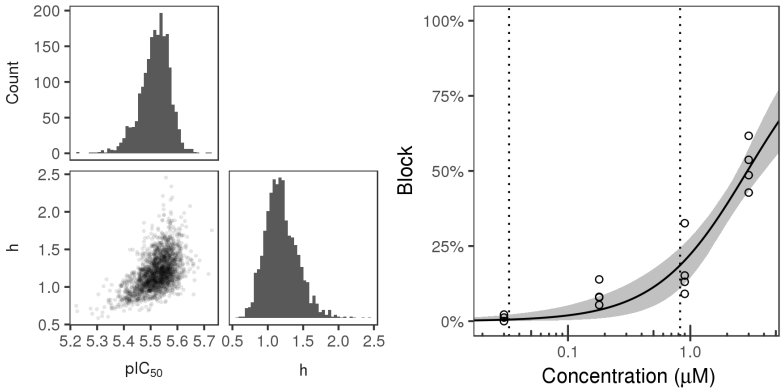


**(A)** Hill equation parameters **(B)** Dose-response curve

**Figure S18.** MCMC simulation results for bepridil-$I_{Na}$ Hill equation parameters.

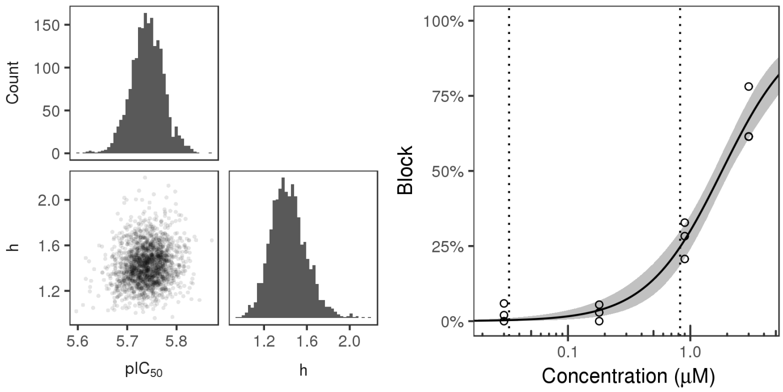


**(A)** Hill equation parameters **(B)** Dose-response curve

**Figure S19.** MCMC simulation results for bepridil-$I_{NaL}$ Hill equation parameters.

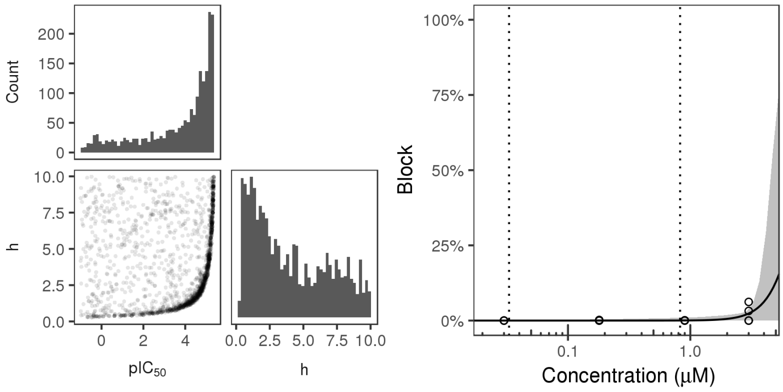


**(A)** Hill equation parameters **(B)** Dose-response curve

**Figure S20.** MCMC simulation results for bepridil-$I_{to}$ Hill equation parameters.

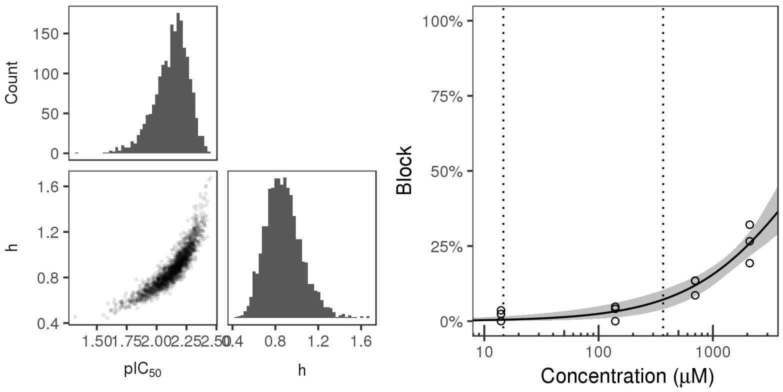


**(A)** Hill equation parameters **(B)** Dose-response curve

**Figure S21.** MCMC simulation results for sotalol-$I_{CaL}$ Hill equation parameters.

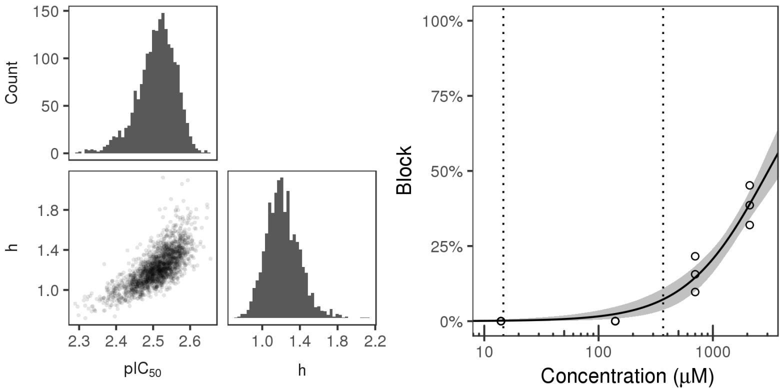


**(A)** Hill equation parameters **(B)** Dose-response curve

**Figure S22.** MCMC simulation results for sotalol-$I_{K1}$ Hill equation parameters.

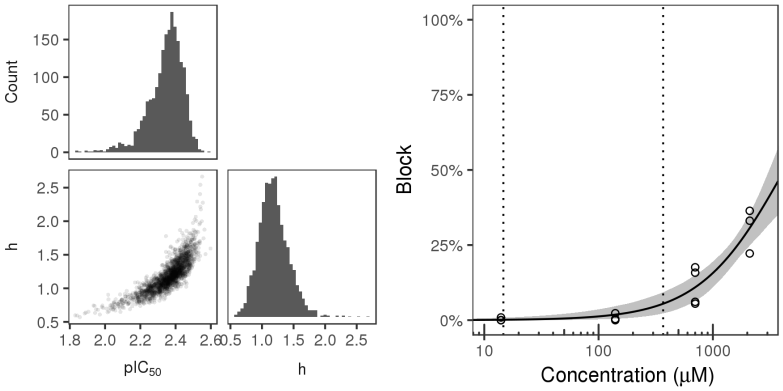


**(A)** Hill equation parameters **(B)** Dose-response curve

**Figure S23.** MCMC simulation results for sotalol-$I_{Ks}$ Hill equation parameters.

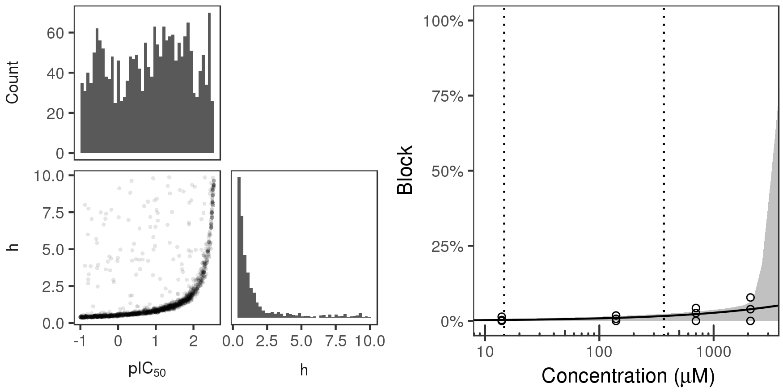


**(A)** Hill equation parameters **(B)** Dose-response curve

**Figure S24.** MCMC simulation results for sotalol-$I_{Na}$ Hill equation parameters.

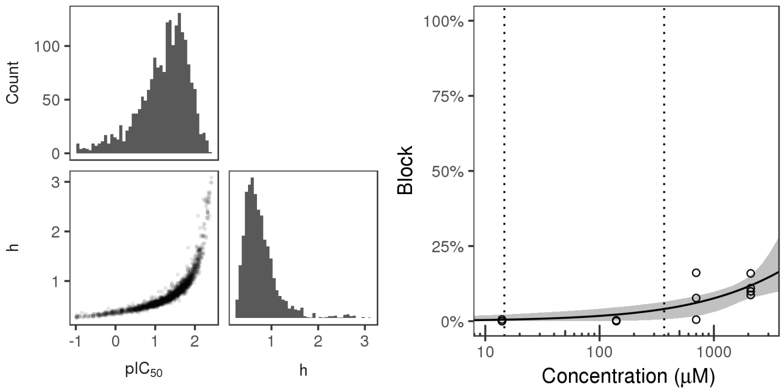


**(A)** Hill equation parameters **(B)** Dose-response curve

**Figure S25.** MCMC simulation results for sotalol-$I_{to}$ Hill equation parameters.

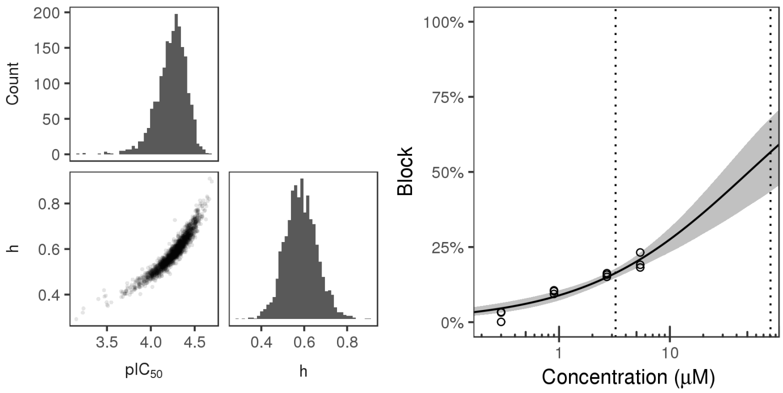


**(A)** Hill equation parameters **(B)** Dose-response curve

**Figure S26.** MCMC simulation results for quinidine-$I_{CaL}$ Hill equation parameters.

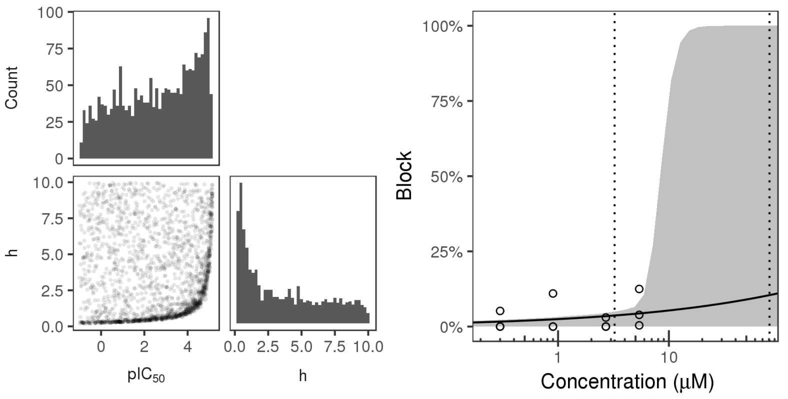


**(A)** Hill equation parameters **(B)** Dose-response curve

**Figure S27.** MCMC simulation results for quinidine-$I_{K1}$ Hill equation parameters.

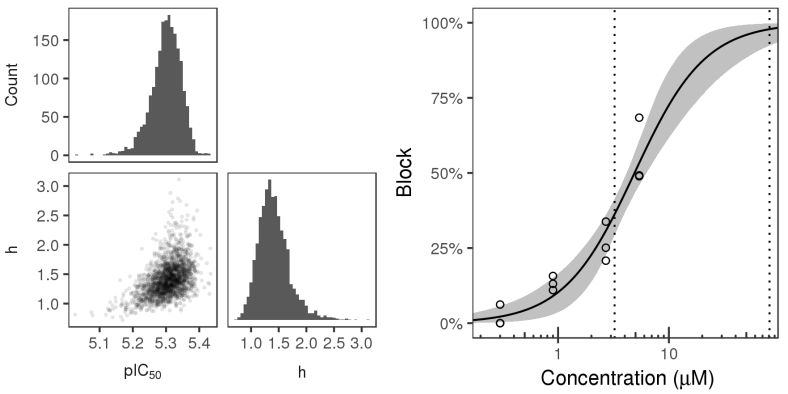


**(A)** Hill equation parameters **(B)** Dose-response curve

**Figure S28.** MCMC simulation results for quinidine-$I_{Ks}$ Hill equation parameters.

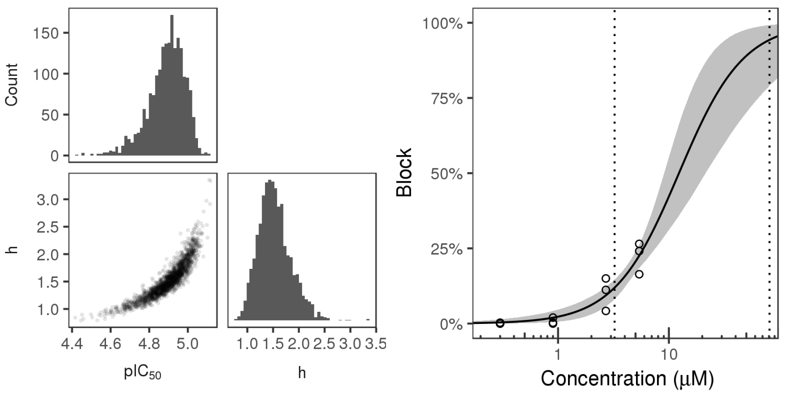


**(A)** Hill equation parameters **(B)** Dose-response curve

**Figure S29.** MCMC simulation results for quinidine-$I_{Na}$ Hill equation parameters.

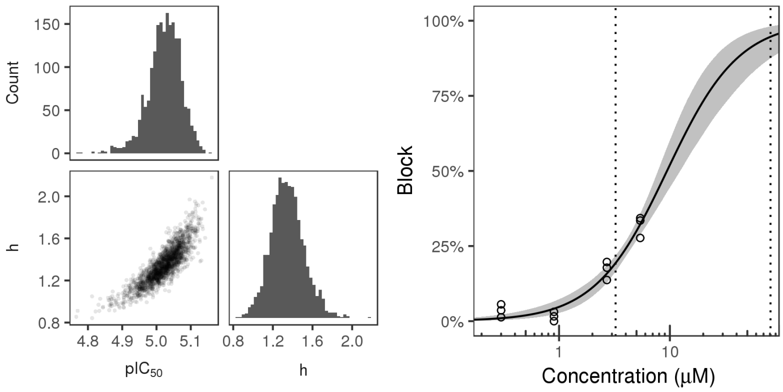


**(A)** Hill equation parameters **(B)** Dose-response curve

**Figure S30.** MCMC simulation results for quinidine-$I_{NaL}$ Hill equation parameters.

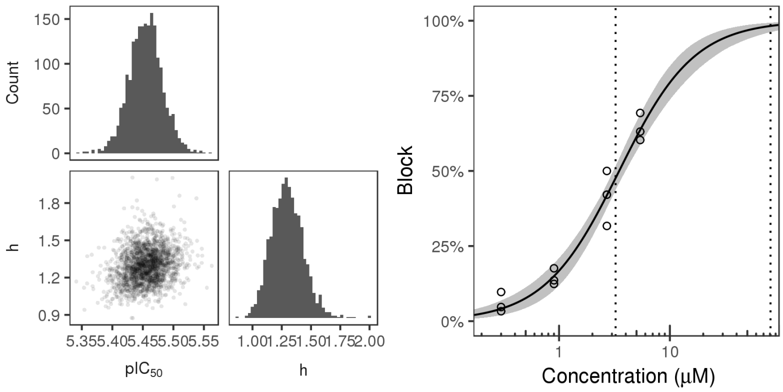


**(A)** Hill equation parameters **(B)** Dose-response curve

**Figure S31.** MCMC simulation results for quinidine-$I_{to}$ Hill equation parameters.

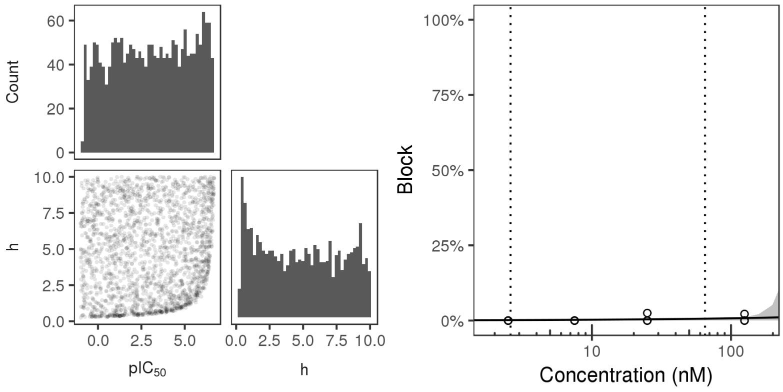


**(A)** Hill equation parameters **(B)** Dose-response curve

**Figure S32.** MCMC simulation results for cisapride-$I_{CaL}$ Hill equation parameters.

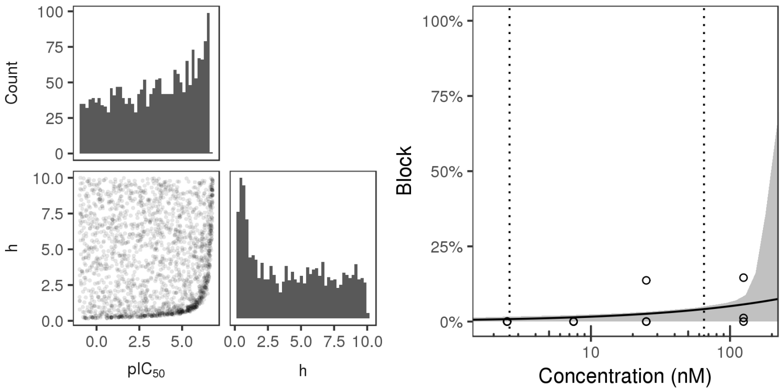


**(A)** Hill equation parameters **(B)** Dose-response curve

**Figure S33.** MCMC simulation results for cisapride-$I_{K1}$ Hill equation parameters.

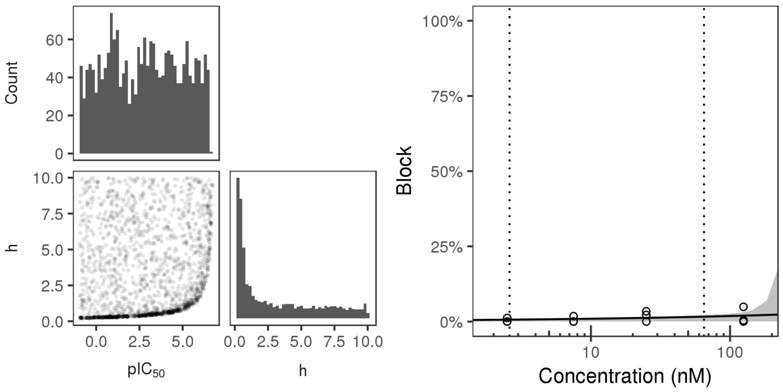


**(A)** Hill equation parameters **(B)** Dose-response curve

**Figure S34.** MCMC simulation results for cisapride-$I_{Ks}$ Hill equation parameters.

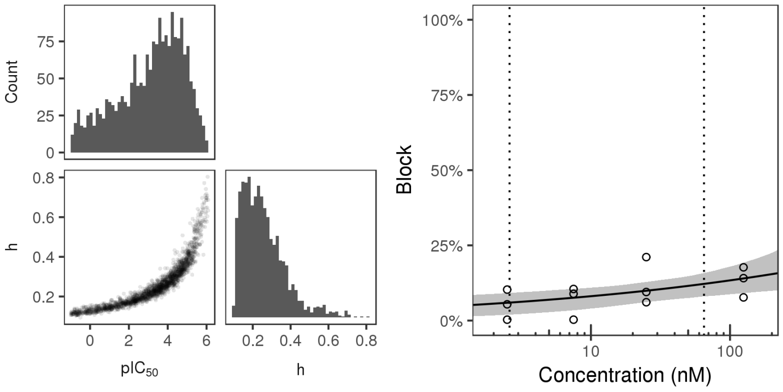


**(A)** Hill equation parameters **(B)** Dose-response curve

**Figure S35.** MCMC simulation results for cisapride-$I_{to}$ Hill equation parameters.

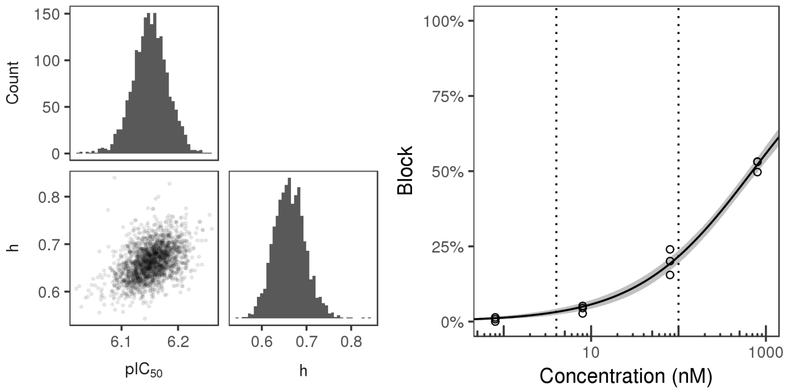


**(A)** Hill equation parameters **(B)** Dose-response curve

**Figure S36.** MCMC simulation results for terfenadine-$I_{CaL}$ Hill equation parameters.

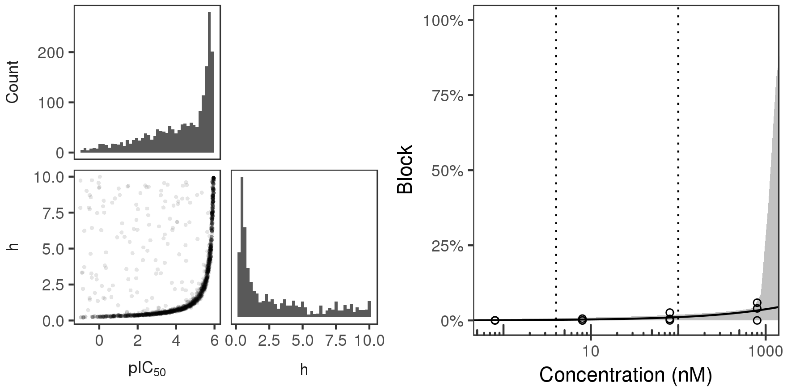


**(A)** Hill equation parameters **(B)** Dose-response curve

**Figure S37.** MCMC simulation results for terfenadine-$I_{Ks}$ Hill equation parameters.

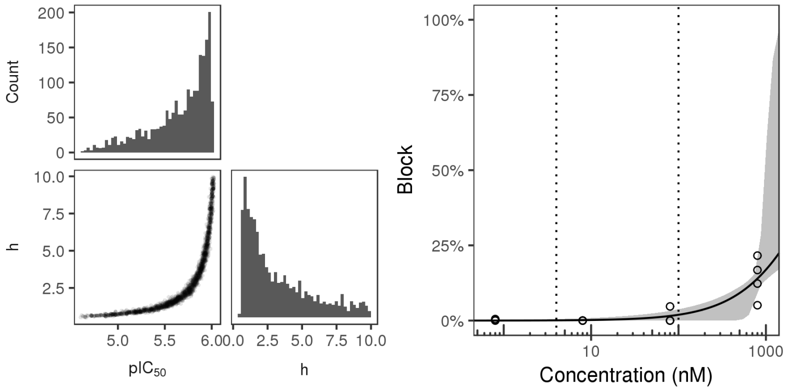


**(A)** Hill equation parameters **(B)** Dose-response curve

**Figure S38.** MCMC simulation results for terfenadine-$I_{Na}$ Hill equation parameters.

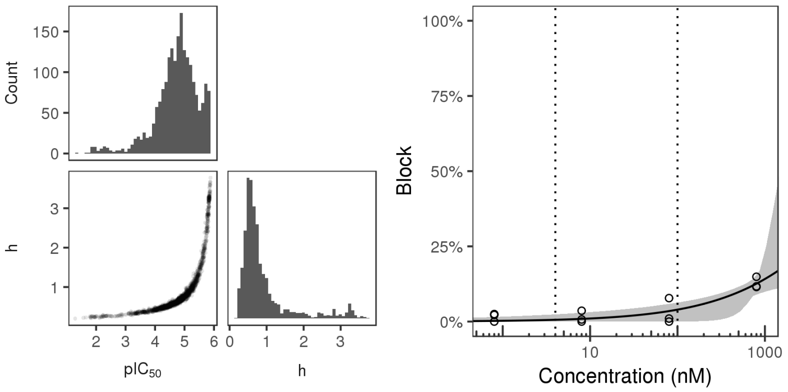


**(A)** Hill equation parameters **(B)** Dose-response curve

**Figure S39.** MCMC simulation results for terfenadine-$I_{NaL}$ Hill equation parameters.

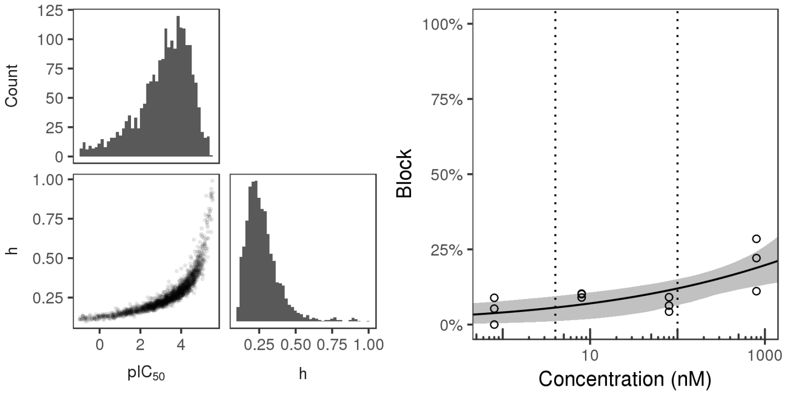


**(A)** Hill equation parameters **(B)** Dose-response curve

**Figure S40.** MCMC simulation results for terfenadine-$I_{to}$ Hill equation parameters.

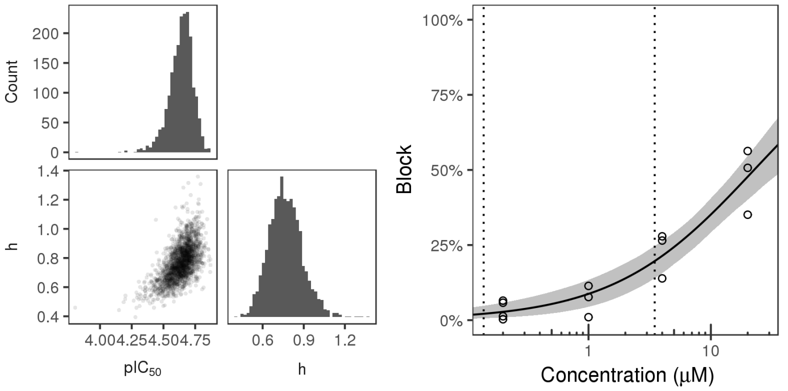


**(A)** Hill equation parameters **(B)** Dose-response curve

**Figure S41.** MCMC simulation results for ondansetron-$I_{CaL}$ Hill equation parameters.

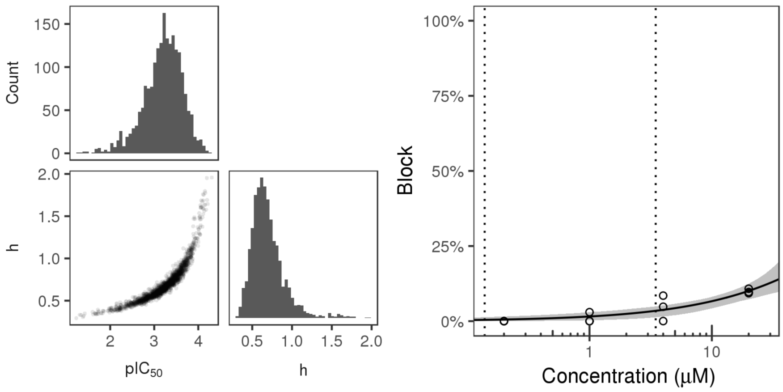


**(A)** Hill equation parameters **(B)** Dose-response curve

**Figure S42.** MCMC simulation results for ondansetron-$I_{Ks}$ Hill equation parameters.

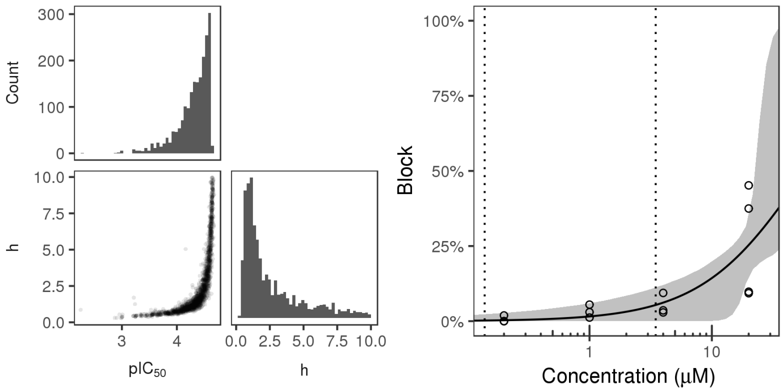


**(A)** Hill equation parameters **(B)** Dose-response curve

**Figure S43.** MCMC simulation results for ondansetron-$I_{Na}$ Hill equation parameters.

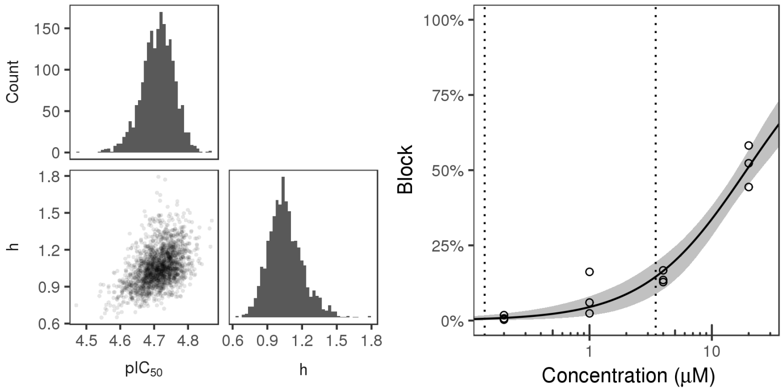


**(A)** Hill equation parameters **(B)** Dose-response curve

**Figure S44.** MCMC simulation results for ondansetron-$I_{NaL}$ Hill equation parameters.

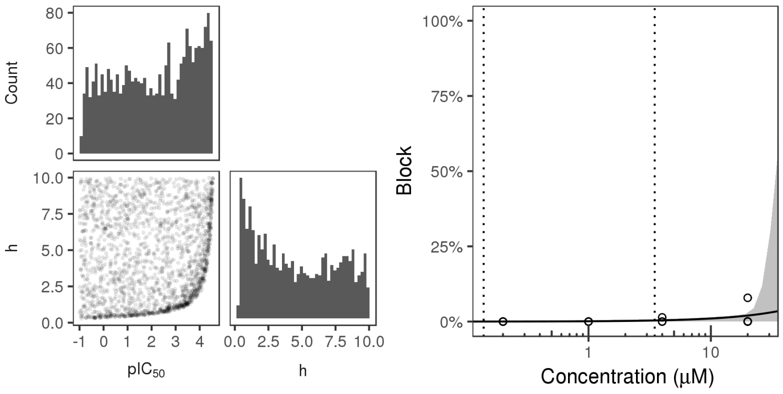


**(A)** Hill equation parameters **(B)** Dose-response curve

**Figure S45.** MCMC simulation results for ondansetron-$I_{to}$ Hill equation parameters.

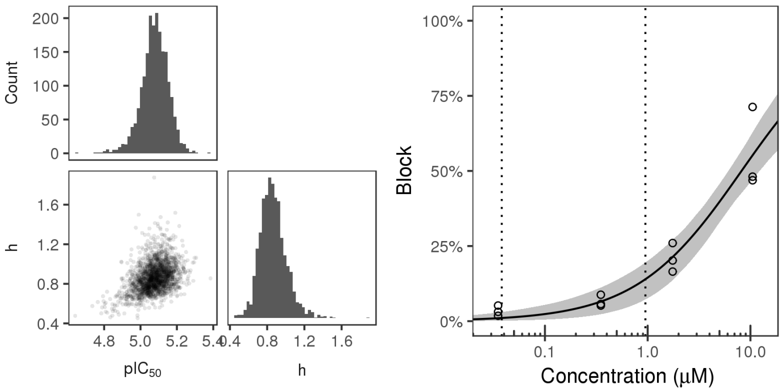


**(A)** Hill equation parameters **(B)** Dose-response curve

**Figure S46.** MCMC simulation results for chlorpromazine-$I_{CaL}$ Hill equation parameters.

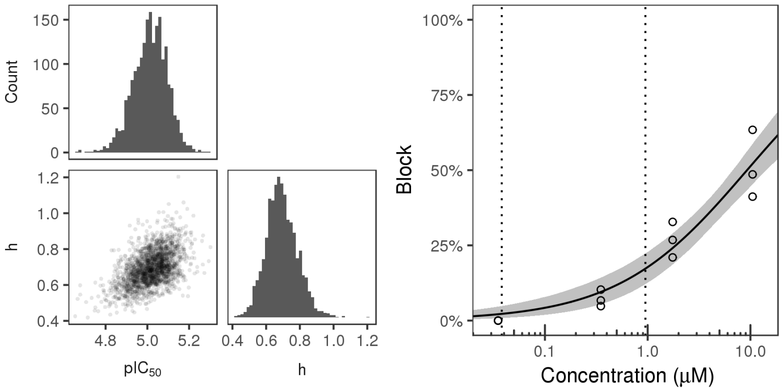


**(A)** Hill equation parameters **(B)** Dose-response curve

**Figure S47.** MCMC simulation results for chlorpromazine-$I_{K1}$ Hill equation parameters.

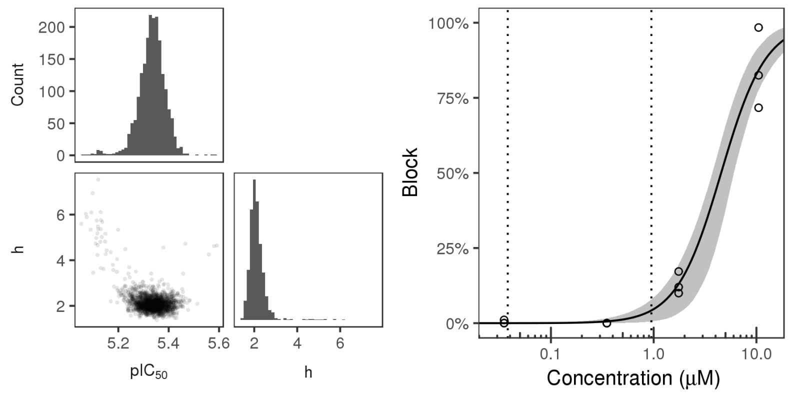


**(A)** Hill equation parameters **(B)** Dose-response curve

**Figure S48.** MCMC simulation results for chlorpromazine-$I_{Na}$ Hill equation parameters.

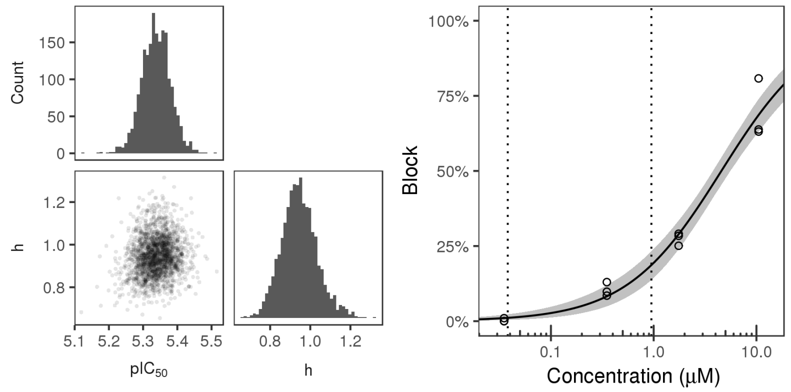


**(A)** Hill equation parameters **(B)** Dose-response curve

**Figure S49.** MCMC simulation results for chlorpromazine-$I_{NaL}$ Hill equation parameters.

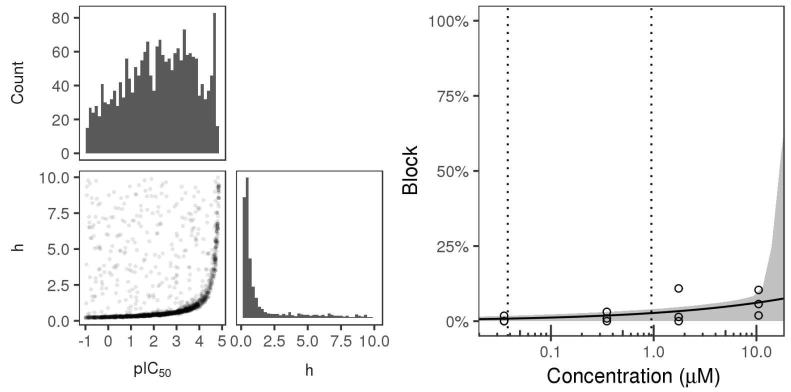


**(A)** Hill equation parameters **(B)** Dose-response curve

**Figure S50.** MCMC simulation results for chlorpromazine-$I_{to}$ Hill equation parameters.

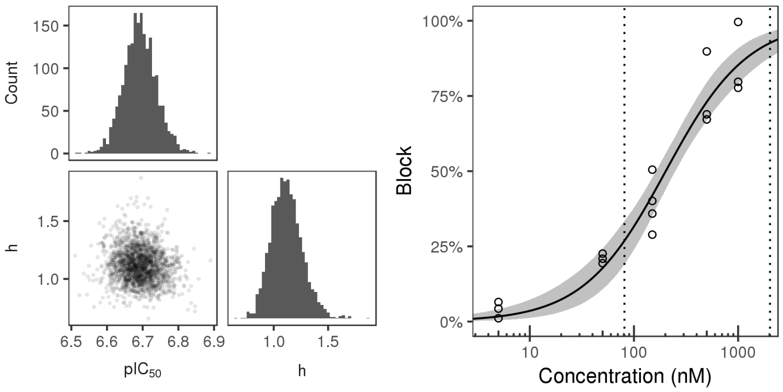


**(A)** Hill equation parameters **(B)** Dose-response curve

**Figure S51.** MCMC simulation results for verapamil-$I_{CaL}$ Hill equation parameters.

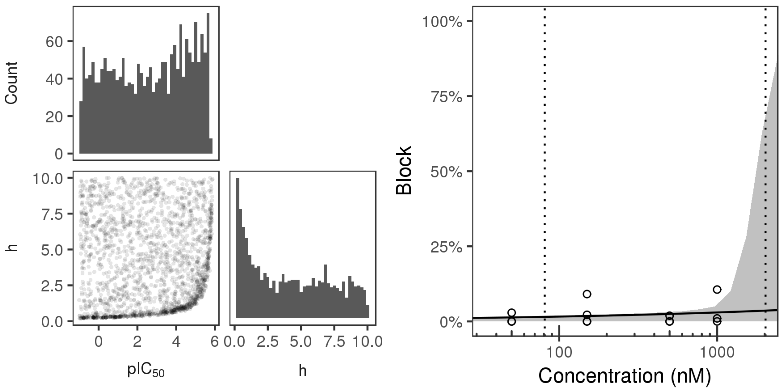


**(A)** Hill equation parameters **(B)** Dose-response curve

**Figure S52.** MCMC simulation results for verapamil-$I_{K1}$ Hill equation parameters.

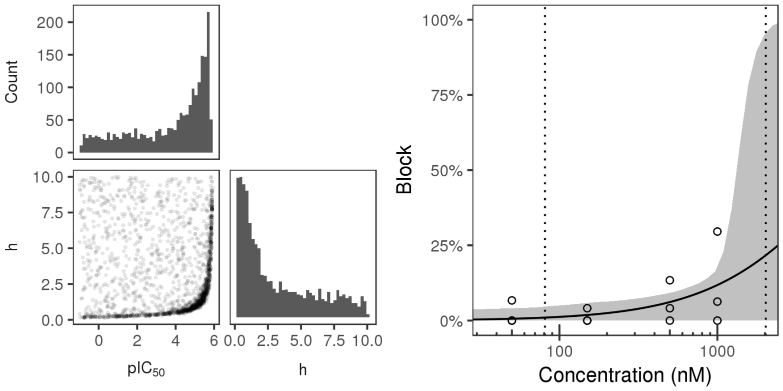


**(A)** Hill equation parameters **(B)** Dose-response curve

**Figure S53.** MCMC simulation results for verapamil-$I_{NaL}$ Hill equation parameters.

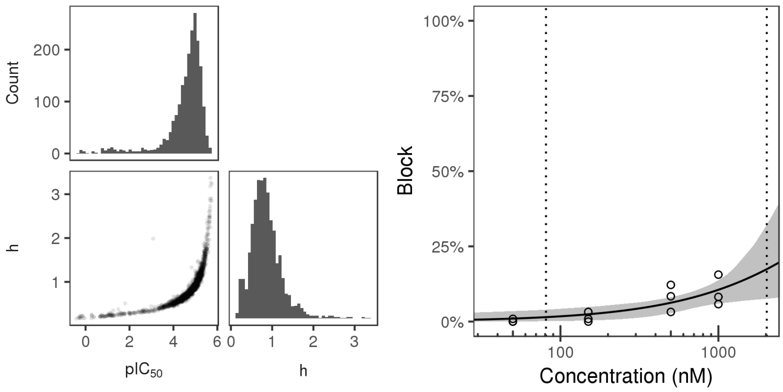


**(A)** Hill equation parameters **(B)** Dose-response curve

**Figure S54.** MCMC simulation results for verapamil-$I_{to}$ Hill equation parameters.

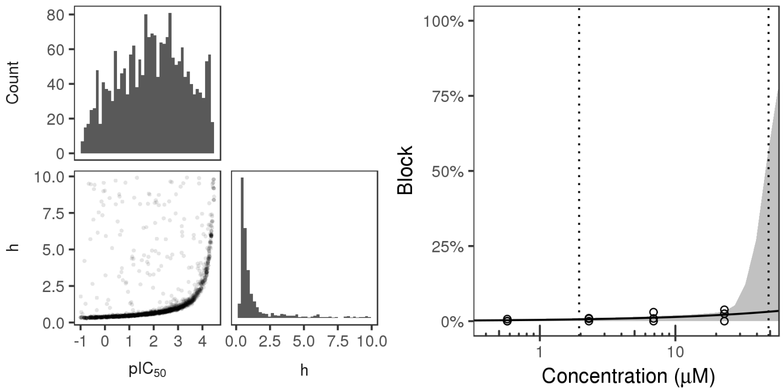


**(A)** Hill equation parameters **(B)** Dose-response curve

**Figure S55.** MCMC simulation results for ranolazine-$I_{Ks}$ Hill equation parameters.

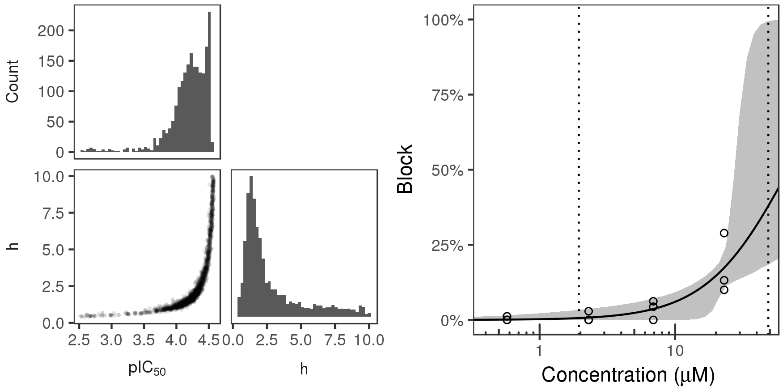


**(A)** Hill equation parameters **(B)** Dose-response curve

**Figure S56.** MCMC simulation results for ranolazine-$I_{Na}$ Hill equation parameters.

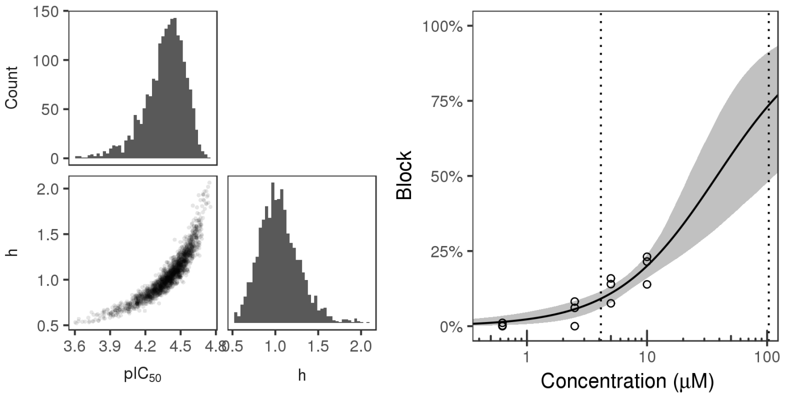


**(A)** Hill equation parameters **(B)** Dose-response curve

**Figure S57.** MCMC simulation results for mexiletine-$I_{CaL}$ Hill equation parameters.

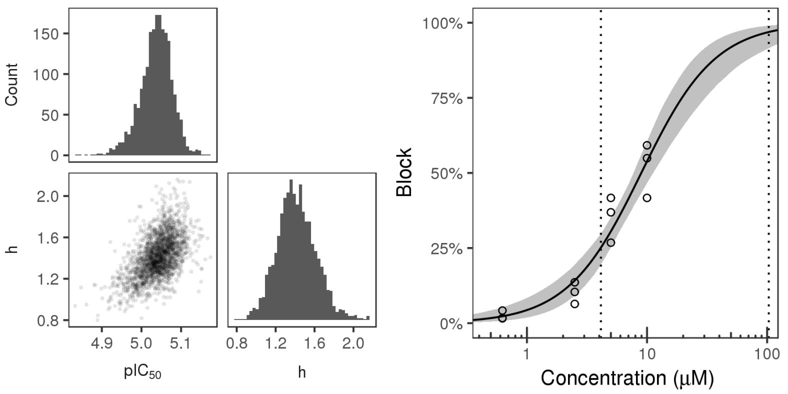


**(A)** Hill equation parameters **(B)** Dose-response curve

**Figure S58.** MCMC simulation results for mexiletine-$I_{NaL}$ Hill equation parameters.

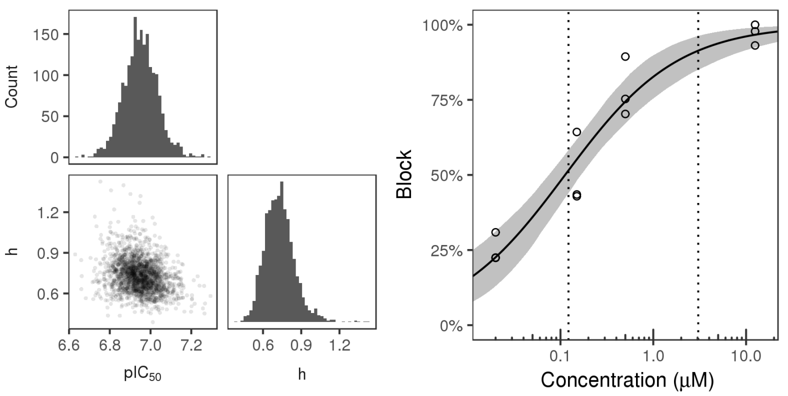


**(A)** Hill equation parameters **(B)** Dose-response curve

**Figure S59.** MCMC simulation results for diltiazem-$I_{CaL}$ Hill equation parameters.

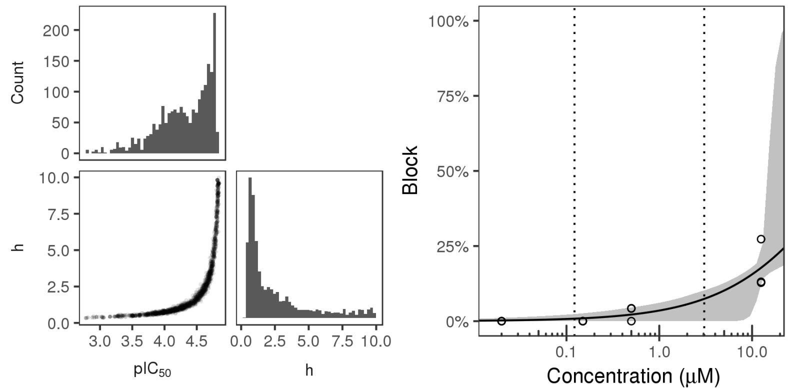


**(A)** Hill equation parameters **(B)** Dose-response curve

**Figure S60.** MCMC simulation results for diltiazem-$I_{Na}$ Hill equation parameters.

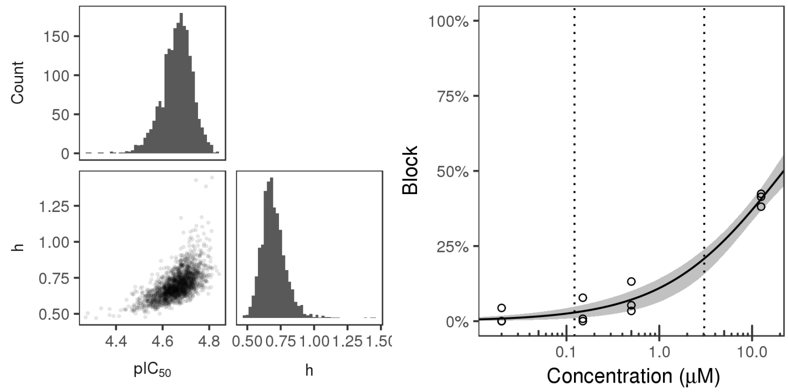


**(A)** Hill equation parameters **(B)** Dose-response curve

**Figure S61.** MCMC simulation results for diltiazem-$I_{NaL}$ Hill equation parameters.

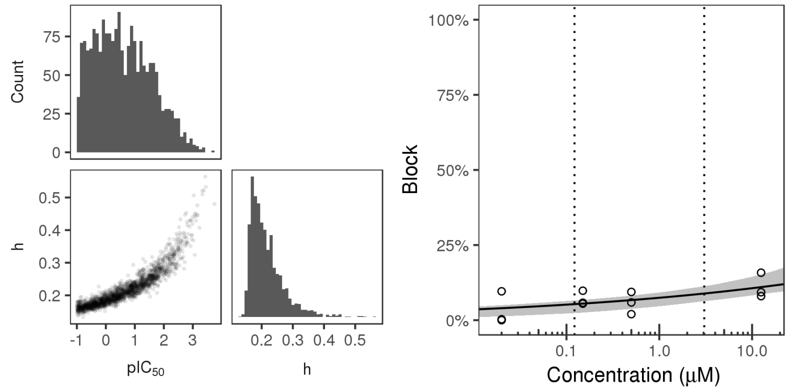


**(A)** Hill equation parameters **(B)** Dose-response curve

**Figure S62.** MCMC simulation results for diltiazem-$I_{to}$ Hill equation parameters.

## REFERENCES

Crumb, J., W. J., Vicente, J., Johannesen, L., and Strauss, D. G. (2016). An evaluation of 30 clinical drugs against the comprehensive in vitro proarrhythmia assay (CiPA) proposed ion channel panel. *J Pharmacol Toxicol Methods* 81, 251–62. doi:10.1016/j.vascn.2016.03.009

Dutta, S., Chang, K., Beattie, K., Sheng, J., Tran, P. N., Wu, W. W., et al. (2017). Optimization of an in silico cardiac cell model for proarrhythmia risk assessment. *Frontiers in Physiology*

Geweke, J. (1992). Evaluating the accuracy of sampling-based approaches to the calculation of posterior moments. In *Bayesian Statistics 4*, eds. J. M. Bernardo, J. O. Berger, A. P. Dawid, and A. F. M. Smith (Clarendon Press). 1 edn., 169–193

Hansen, N. (2006). The cma evolution strategy: A comparing review. In *Towards a New Evolutionary Computation: Advances in the Estimation of Distribution Algorithms*, eds. J. A. Lozano, P. Larraaga, I. Inza, and E. Bengoetxea (Berlin, Heidelberg: Springer Berlin Heidelberg). 75–102. doi:10.1007/3-540-32494-1_4

Kramer, J., Obejero-Paz, C. A., Myatt, G., Kuryshev, Y. A., Bruening-Wright, A., Verducci, J. S., et al. (2013). MICE models: superior to the HERG model in predicting Torsade de Pointes. *Sci Rep* 3, 2100. doi:10.1038/srep02100

Li, Z., Dutta, S., Sheng, J., Tran, P. N., Wu, W., Chang, K., et al. (2017). Improving the in silico assessment of proarrhythmia risk by combining hERG (Human Ether-à-go-go-Related Gene) channel-drug binding kinetics and multichannel pharmacology. *Circulation: Arrhythmia and Electrophysiology* 10, e004628. doi:10.1161/circep.116.004628

Milnes, J. T., Witchel, H. J., Leaney, J. L., Leishman, D. J., and Hancox, J. C. (2010). Investigating dynamic protocol-dependence of herg potassium channel inhibition at 37 degrees C: Cisapride versus dofetilide. *J Pharmacol Toxicol Methods* 61, 178–91. doi:10.1016/j.vascn.2010.02.007

Plummer, M., Best, N., Cowles, K., and Vines, K. (2006). CODA: Convergence diagnosis and output analysis for MCMC. *R News* 6, 7–11

Redfern, W., Carlsson, L., Davis, A., Lynch, W., Mackenzie, I., Palethorpe, S., et al. (2003). Relationships between preclinical cardiac electrophysiology, clinical qt interval prolongation and torsade de pointes for a broad range of drugs: evidence for a provisional safety margin in drug development. *Cardiovascular Research* 58, 32–45. doi:10.1016/s0008-6363(02)00846-5

Soetaert, K. and Petzoldt, T. (2010). Inverse modelling, sensitivity and Monte Carlo analysis in R using package FME. *Journal of Statistical Software* 33, 1–28. doi:10.18637/jss.v033.i03

Trautmann, H., Mersmann, O., and Arnu, D. (2011). cmaes: Covariance matrix adapting evolutionary strategy. R package version 1.0-11
